# Human adenovirus type 17 from species D transduces endothelial cells and human CD46 is involved in cell entry

**DOI:** 10.1038/s41598-018-31713-x

**Published:** 2018-09-07

**Authors:** Jing Liu, Philip Boehme, Wenli Zhang, Jun Fu, Roma Yumul, Kemal Mese, Raphael Tsoukas, Manish Solanki, Michael Kaufmann, Ruirui Lu, Achim Schmidtko, A. Francis Stewart, André Lieber, Anja Ehrhardt

**Affiliations:** 1 Institute for Virology and Microbiology, Center for Biomedical Education and Research (ZBAF), Witten/Herdecke University, Witten, Germany; 2 Shandong University-Helmholtz Institute of Biotechnoloy, State Key Laboratory of Microbial Technology, School of Life Science, Shandong University, Jinan, 250100 People’s Republic of China; 3 Genomics, Biotechnology Center, Technische Universität Dresden, BioInnovations Zentrum, Dresden, Germany; 4 Division for Medical Biochemistry, Center for Biomedical Education and Research (ZBAF), Witten/Herdecke University, Witten, Germany; 5 University of Washington, Department of Medicine, Division of Medical Genetics, Seattle, USA; 6 Institute for Pharmakology and Toxicology, Center for Biomedical Education and Research (ZBAF), Witten/Herdecke University, Witten, Germany; 7 Present Address: Department of Oncology and cancer immunotherapy, University of Maryland School of Medicine, Baltimore, MD USA; 8 Present Address: Medical Student, Department of Human Medicine, Faculty of Health, Witten/Herdecke University, Witten, Germany; 9 Present Address: Institute of Pharmacology, College of Pharmacy, Goethe University, Frankfurt am Main, Germany; 10 Present Address: Institute for Experimental Gene Therapy and Cancer Research (IEGT), Medical University Rostock, Rostock, Germany

## Abstract

More than 70 human adenoviruses with type-dependent pathogenicity have been identified but biological information about the majority of these virus types is scarce. Here we employed multiple sequence alignments and structural information to predict receptor usage for the development of an adenoviral vector with novel biological features. We report the generation of a cloned adenovirus based on human adenovirus type 17 (HAdV17) with high sequence homology to the well characterized human adenovirus type 37 (HAdV37) that causes epidemic keratoconjunctivitis (EKC). Our study revealed that human CD46 (CD46) is involved in cell entry of HAdV17. Moreover, we found that HAdV17 infects endothelial cells (EC) *in vitro* including primary cells at higher efficiencies compared to the commonly used human adenovirus type 5 (HAdV5). Using a human CD46 transgenic mouse model, we observed that HAdV17 displays a broad tropism *in vivo* after systemic injection and that it transduces ECs in this mouse model. We conclude that the HAdV17-based vector may provide a novel platform for gene therapy.

## Introduction

Human adenoviruses are non-enveloped and double-stranded DNA viruses. To date more than 70 human adenovirus types (HAdV) have been identified^1^, which are classified into seven species (A-G) according to hemagglutination and serum neutralization capacities. HAdV-B, -D members cause disease in the eyes, HAdV-A, -B, -C, -E mainly infect airways and HAdV-F,G predominantly display a gastrointestinal tropism^2,3^. The tropism of human adenoviruses is in part defined by their attachment to the respective cell surface receptor to its capsid fiber protein. HAdV5 and 2 from species C use CAR as primary attachment receptor, but there is also evidence that HAdVs from other species (e.g. A, E, and F) can bind to CAR^4^. In contrast adenoviruses from species B that cause ocular, respiratory or urinary tract infections utilize CD46 or desmoglein-2 (DSG-2) were described as cellular binding structures^5,6^. Some species D adenovirus types such as types 9, 10, and 24 can also use CAR as primary receptor^4^, but some types from species D (types 8, 19a, 37) which cause epidemic keratoconjunctivitis can bind to sialic acid and glycans^7^. Moreover, there are hints that other members of species D (e.g. types 26, 48, 49) can also bind to CD46 as receptor^8–10^. However, it remains to be shown whether CD46 is a primary receptor for these viruses and especially for type 48 conflicting information exists^11,12^.

This type-dependent tropism and the large number of different HAdVs makes adenovirus attractive for therapeutic applications in biomedicine such as gene therapy, oncolytic virotherapy, and vaccination. HAdV5 represents the most widely used vector combining the capability of delivering large transgene cassettes with efficient transduction of a broad range of dividing and quiescent cells. However, previous studies highlighted several disadvantages of the HAdV5-based vector system including the stimulation of strong innate and adoptive immune responses and the predefined natural tropism that prevents efficient transduction of HAdV5 resistant cell types^13,14^. Moreover pre-existing neutralizing antibodies in up to 90% of the human population can eliminate transduced cells^15,16^. Due to these disadvantages, genetic or chemical capsid modifications have been applied as ways to improve features of conventionally used HAdV5 vectors^17,18^. Another option is to develop alternative vectors based on different adenovirus types that might have more suitable properties in gene transfer applications.

Most studies of human adenoviruses have been based on HAdV5 and a handful of other serotypes because genetic access to other adenovirus types has been difficult. To bypass this bottleneck we recently developed new methods to clone and engineer new adenoviral isolates^19^. Thereby we established a novel library of cloned adenovirus genomes which will facilitate a systematic exploration of the complete spectrum of adenoviruses to study pathogenesis and biomedical approaches. To begin this study, we chose HAdV17, which was, first isolated from conjunctival scrapings in 1955^20^ and is derived from the largest group of species D adenoviruses, because its infection biology and tropism are largely unknown. HAdV17 shows highest sequence homology to the pathogenic adenovirus type 37 (HAdV37) causing EKC with highest amino acid similarity (72%) to HAd17. It was shown that HAdV37 utilizes CD46^21^ and GD1a glycan^7^ as cellular receptors and that this virus has low binding affinities to CAR^22^. An earlier study demonstrated that wild type HAdV17 can efficiently infect airway epithelial cells^23^ and it was speculated that it may use CAR as cellular receptor^23^. Using multiple sequence alignments and available structure information, we predicted receptor usage and developed an adenoviral vector with novel biological features. Gathered information suggested that HAdV17 can use CD46 as cell surface binding structure. Furthermore, after performing a cellular screen we established that HAdV17 shows increased transduction efficiencies of endothelial cells *in vitro* if directly compared to HAdV5.

## Results

### Structure-based predictions of adenovirus receptor usage

**Figure 1:**
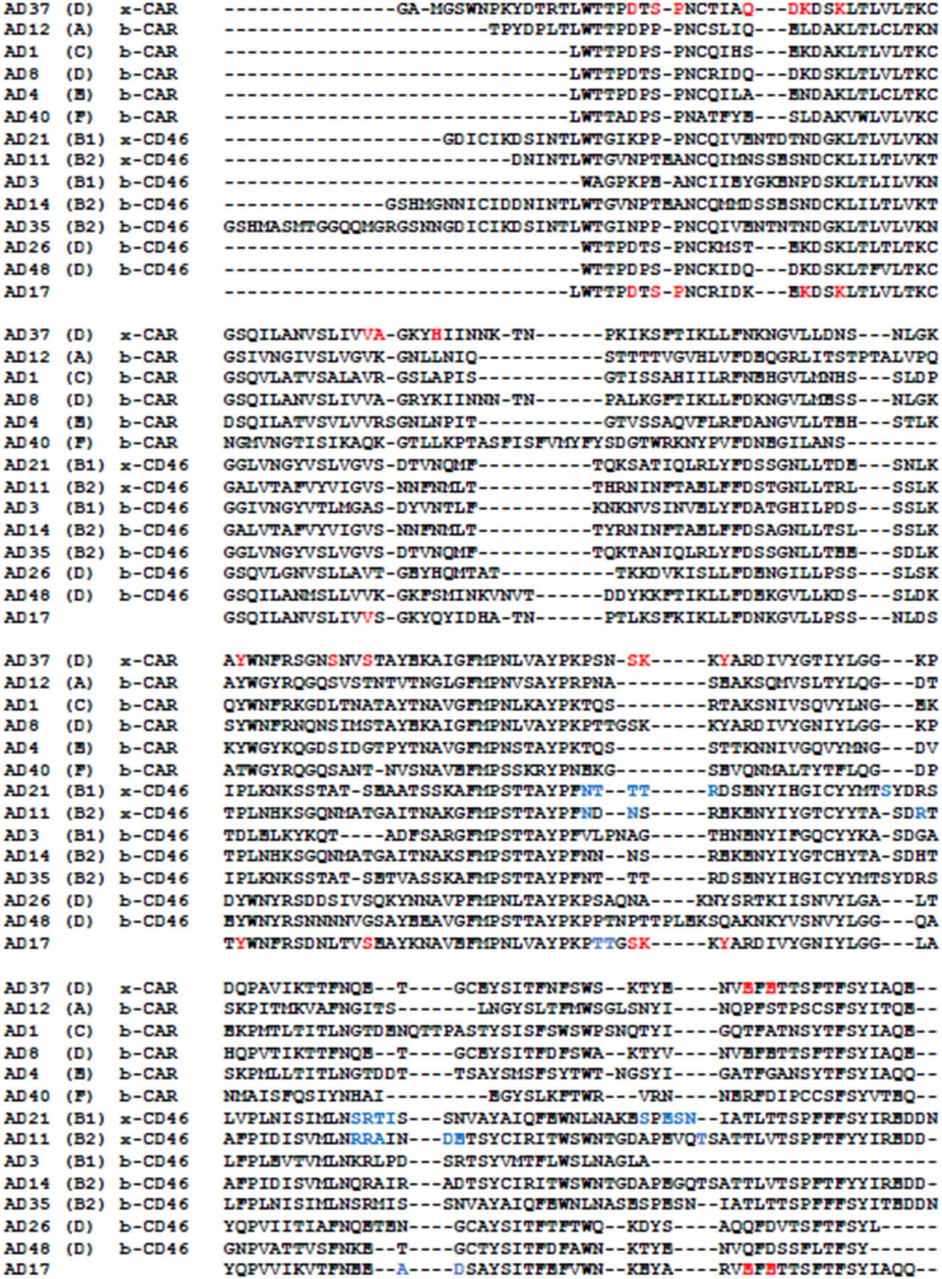
Multiple sequence alignment of fiber knob protein sequences from different adenoviruses. Amino acids known to interact with CAR by X-ray diffraction experiments^24^ are colored red and those known to interact with CD46 by X-ray diffraction experiments^25,26^ are colored blue. Proteins known to bind to CAR or CD46 are indicated by b-CAR and b-CD46^27,28^ and those whose binding is established by X-ray diffraction experiments are indicated by x-CAR and x-CD46^26–28^. The adenovirus species is shown in brackets.

It is known that CAR can be used for cell entry by several human adenoviruses species and that CD46 is used as a cell surface attachment structure mainly by members of species B adenoviruses. Species D adenoviruses seem to display diverse binding affinities and binding modes to known adenovirus binding structures on the cell surface. The first contact with virus with the target cell is initiated by the fiber knob protruding from the capsid. Here we hypothesized that receptor predictions can be based on multiple sequence alignments and known x-ray structures^24–28^. For that purpose multiple sequence alignments were performed with (i) HAdV17, (ii) fiber knob proteins with resolved x-ray structures in complex with CAR as well as CD46, and (iii) fiber knob proteins from different serotypes known to bind CAR or CD46. Amino acids of fiber knob proteins known to be involved in receptor binding from X-ray structures were compared to the corresponding ones within the HAdV17 sequence. As shown in Fig. 1, 13 amino acids described to interact with CAR (red) and 4 amino acids known to interact with CD46 (blue) are also present within the AD17 sequence suggesting that HAdV17 might bind to both, CD46 and CAR host receptors.

### HAdV17 displays an enhanced endothelium tropism *in vitro* compared to HAdV5

**Figure 2:**
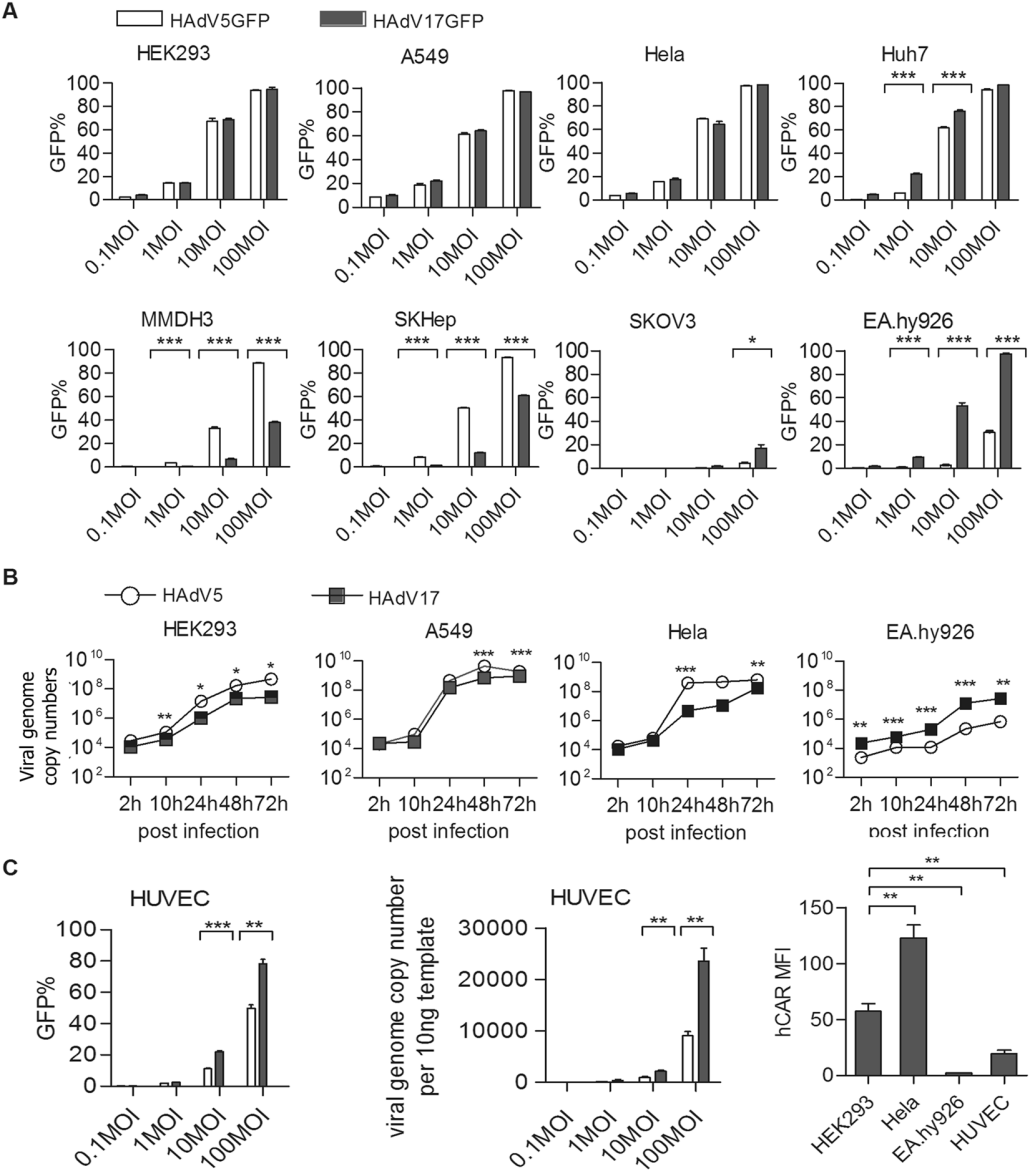
HAdV17 has tropism for endothelium cells *in vitro*. **(A)** Cell line screening *in vitro*. HEK293-, A549-, Hela-, Huh7-, MMDH3-, SKHep-, SKOV3-, and EA.hy926 cells were transduced with HAdV5GFP and HAdV17GFP at various MOIs (0.1, 1, 10 or 100). GFP expression levels were analyzed 24 hrs post-infection by FACS analyses. Uninfected cells (negative controls) were used to set the background gate at approximately 1%. Percentage provided indicates % of GFP-positive cells. A total of 10,000 viable cells were counted. **(B)** Growth curve comparison between wild type HAdV17 and HAdV5. HEK293-, A549-, Hela and EA.hy926 cells were transduced with wild type HAdV5 and HAdV17 at MOI 10. Cells were harvested at various time points (2, 10, 24, 48, 72 hrs post infection) and qPCR was performed to monitor virus genome copy numbers. **(C)** Transduction of primary human umbilical vein cells (HUVEC). Left panel: Primary HUVECs were transduced with HAdV5GFP and HAdV17GFP at various multiplicities of infection (MOIs 0.1, 1, 10 or 100). GFP-positive cell percentages were analyzed 24 hrs post-infection by FACS analyses. Middle panel: Virus internalization analysis by qPCR. HUVEC cells were infected 2 hrs post-infection internalized viral genomes were analyzed. Data points represent mean standard error out of 3 independent experiments (n = 3). Right panel: Quantification of mean fluorescent intensity of CAR expression on cell surfaces. Hela-, EA.hy926- and HUVEC cells were stained with an anti-CAR antibody labeled with FITC and measured by flow cytometry. As negative controls each cell line was also incubated without supplementation of the primary antibody. Data points represent mean standard error (n = 3). **P < 0.01, ***P < 0.001.

To gather information about HAdV17 tropism, virus-host interactions, pathogenesis, and immune responses, we directly cloned a complete HAdV17 genome from a clinical isolate, and engineered it to a first generation vector by replacing its E1 gene with an eGFP reporter gene. By using a novel homologous recombineering pipeline based on ccdB counter-selection^29^ and the low copy plasmid p15A^19^, we successfully generated the E1-deficient HAdV17-based vector p15A-HAdV17GFP and the E1-deficient p15A-HAdV5GFP vector based on HAdV5 as control (Supplementary Fig. 1). HAdV5GFP was reconstituted in HEK293 cells carrying the left arm of the HAdV5 genome including the early E1 gene. To rescue HAdV17GFP a modified complementation cell line based on HEK293 cells was produced that stably expresses the whole E1 gene from HAdV17 (Supplementary Fig. 2). This stable complementary cell line was sufficient to amplify HAdV17GFP including large-scale amplification followed by CsCl gradient centrifugation and dialysis to purify respective viruses. We obtained comparable titers from both virus preparations. To analyze the tropism of HAdV17 *in vitro* we screened a variety of cell lines from different origin. We directly compared transduction efficiencies of HAdV17GFP and HAdV5GFP in HEK293, A549, HeLa, EA.hy926, Huh7, SKOV3, SKHep-1 and MMDH3 cells (Fig. 2A). We found that transduction efficiencies were comparable in HEK293-, Hela-, and A549 cells, while HAdV5GFP showed higher transduction efficiencies in MMDH3 cells (a differentiated murine hepatocyte cell line) and in SKHep-1 cells (human liver adenocarcinoma cells) (Fig. 2A). Interestingly, HAdV17GFP showed robust transduction of EA.hy926 cells (an immortalized human EC line). This was in contrast to HAdV5GFP displaying low transduction rates in the same cell line. A difference was also observed in SKOV3 cells (human epithelial ovarian carcinoma cells) and to a lesser extend in Huh7 cells (derived from a human hepatocellular carcinoma), which were more susceptible to HAdV17GFP (Fig. 2A). For raw data related to the mean fluorescence intensity (MFI) please refer to Supplementary Fig. 3.

Several events in the adenovirus life cycle might account for the differences in GFP expression among cell lines, including 1) primary attachment and internalization, 2) intracellular trafficking and nuclear transport, 3) replication, transcription and translation, 4) assembly and release. Here we compared growth curves of wild type HAdV17 and HAdV5 in these cell lines. As shown in Fig. 2B, HAdV17 was superior to HAdV5 in the human EC line EA.hy926 and we measured up to 10-fold enhanced genome replication.

As EA.hy926 cells are derived from the primary human umbilical vein cell line HUVEC, we further checked the susceptibility of primary HUVEC cells to HAdV17GFP and HAdV5GFP infection. Similar to EA.hy926 cells, we observed that HAdV17GFP showed significantly higher transduction efficiencies in HUVEC cells, as indicated by transgene expression levels (Fig. 2C, left panel) and levels of genome internalization (Fig. 2C, middle panel). It is well established that HAdV5 uses CAR as its high affinity receptor and as we found that EA.hy926-cells were refractory to HAdV5 infection, we further analyzed CAR expression levels on these cells. Results revealed that HEK293- and Hela cells had higher CAR expression levels as EA.hy926- and HUVEC cells. However, HUVEC cells expressed more CAR molecules than EA.hy926 cells (Fig. 2C, right panel). These results indicated that HAdV17 transduces endothelial cells *in vitro* for which CAR is not the major receptor responsible for uptake.

### HAdV17 can utilize CD46 to enter cells *in vitro*

Because CAR was not essential for HAdV17GFP to infect endothelial cells in our study we investigated whether CD46, could be responsible for the tropism for HAdV17GFP to endothelial cells. Firstly, we confirmed that EA.hy926 cells express CD46 at a comparable level as Hela cells (Supplementary Fig. 4). We found that SKOV3- and Huh7 cells are more susceptible to HAdV17GFP rather than HAdV5GFP (Fig. 2A). It was shown that SKOV3 cells are CAR-negative and CD46-postive, and Huh7 cells have higher CAR and CD46 expression than A549 and Hela cells^30,31^, supporting the hypothesis that CD46 plays a role in HAdV17 infection.

**Figure 3:**
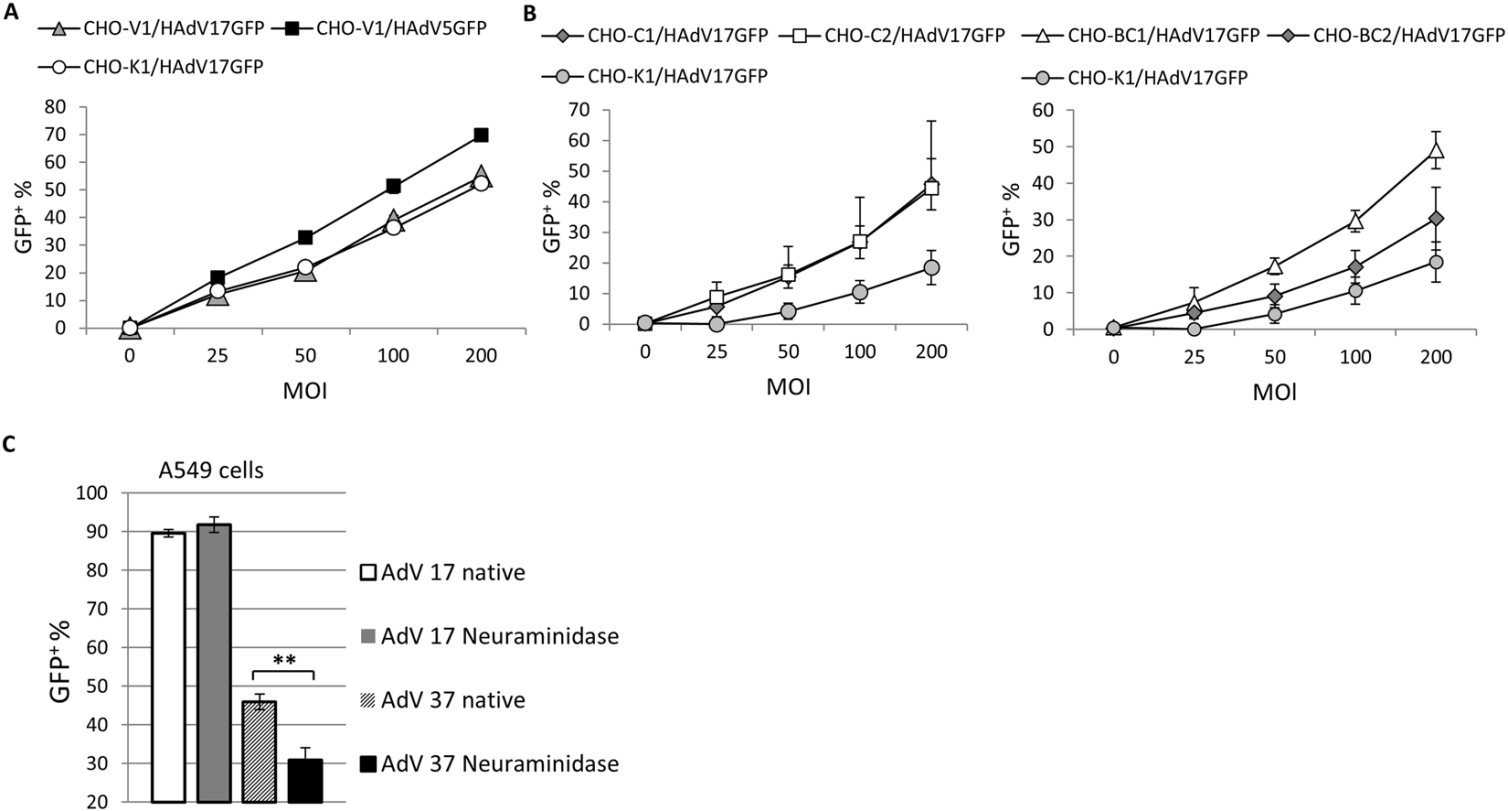
HAdV17 receptor usage. (**A**) GFP positive CHO-V1 cells (CAR positive) determined by flow cytometry after transduction with HAdV5GFP and HAdV17GFP at increasing MOIs as indicated. Percentage provided indicates % of GFP-positive cells. **(B)** Percentage of GFP-positive cells in transduced CD46 positive CHO-C2, CHO-C1, CHO-BC1, CHO-BC2 cells determined by flow cytometry after transduction with HAdV17GFP at increasing MOIs. CHO-K1 cells were used as controls. **(C)** Neuraminidase treated and untreated (native) A549 cells were infected with HAdV17 and HAdV37 and the percentage of GFP positive cells was determined. **P < 0.01.

To further explore potential receptors, we used specific CHO cell lines engineered to express either CAR (CHO-V1) or different CD46 isoforms CD46 (CHO-C1, CHO-C2, CHO-BC1, CHO-BC2)^5^, as well as the CAR/CD46-negative CHO cell line CHO-K1 as control. Cells were infected at various doses (MOIs 25, 50, 100, 150, 200) and results showed that HAdV17GFP could infect either CHO-V1, CHO-C1, CHO-C2 or CHO-BC1 cells at higher efficiencies than CHO-K1 cells (Fig. 3A,B). The transduction ability of HAdV17GFP in CHO-V1 cells was weaker than that of HAdV5GFP (utilizing CAR as receptor) and comparable to the CHO-K1 cell line (Fig. 3A). These results indicated that CD46 could play a role in virus uptake. As another potential receptor we investigated the role of sialic acid for infection with HAdV17. HAdV17 belongs to species D adenoviruses and its sequence is similar to HAdV17 (Fig. 1). It is known that HAdV37 utilizes sialic acid as cellular receptor^32^ and therefore we investigated whether depletion of sialic cells on the cell surface after neuraminidase treatment has an effect on transduction efficiencies of HAdV17. We found that in contrast to HAdV37 neuraminidase treatment had no influence on transduction efficiencies of HAdV17 (Fig. 3C). Therefore, sialic acid can be excluded as potential receptor for HAdV17.

**Figure 4:**
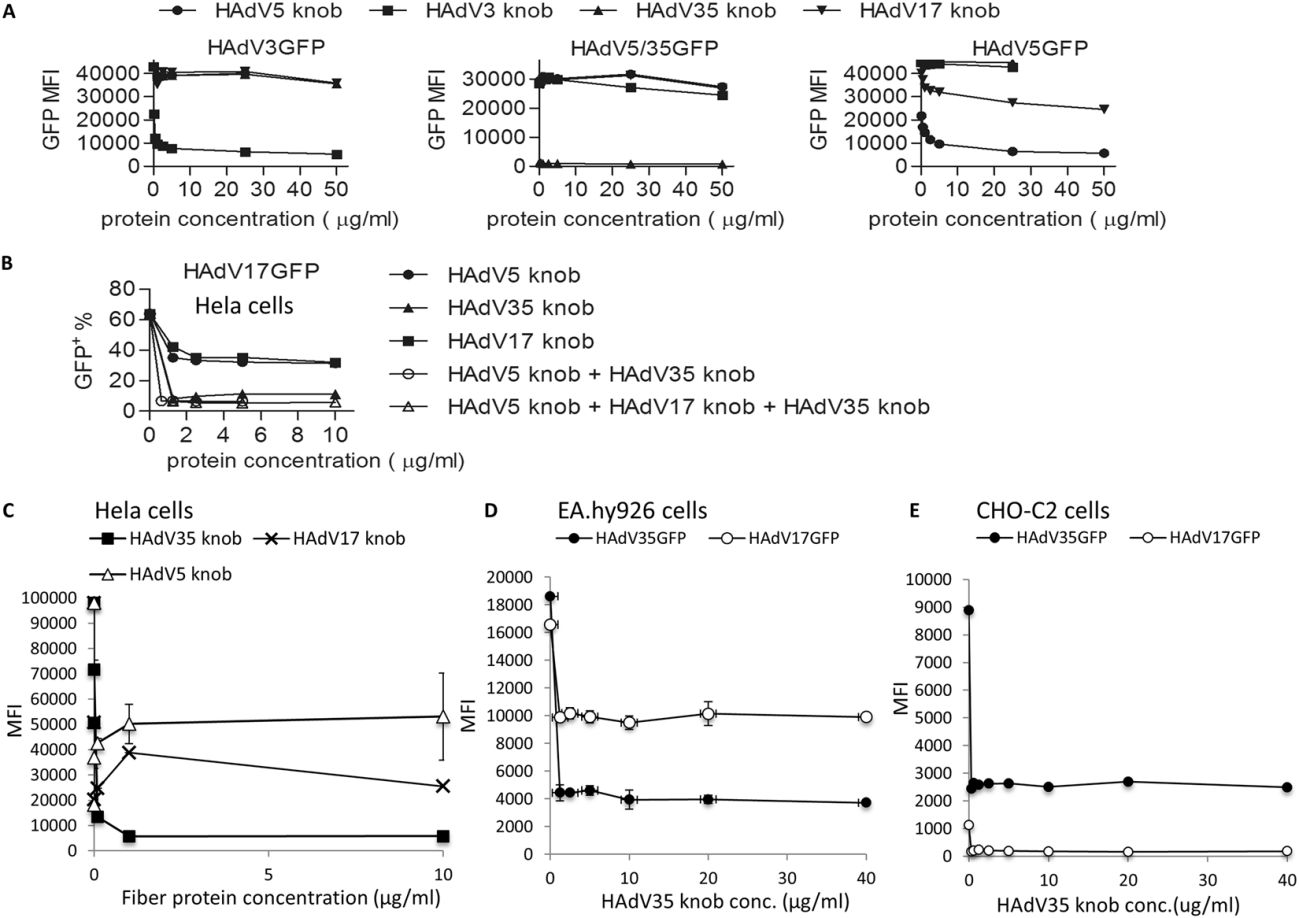
Fiber blocking assays in various cell lines. Adherent Hela cells were pre-incubated with increasing concentrations of recombinant fiber knob proteins (HAdV5 knob, HAdV3 knob, HAdV35 knob, HAdV17 knob or a combination) and then incubated with **(A)** HAdV3GFP, HAdV5/35GFP, and HAdV5GFP, and **(B)** HAdV17GFP. Uninfected cells (negative controls) were used to set the background gate at approximately 1%. MFI indicates mean fluorescence intensity. **(C)** HeLa cells were pretreated with HAdV35 knob, HAdV5 knob and HAdV17 knob at increasing concentrations at 37 °C for 1 hour, and then exposed to HAd17GFP at MOI 100. GFP expression levels (MFI) were measured 24 hrs later by flow cytometry. **(D)** EA.hy926 cells and **(E)** CHO-C2 were pretreated with HAdV35 knob at increasing concentrations at 37 for 1 hour, and then exposed to HAdV17GFP and HAdV35GFP virus at MOI 100. GFP expression (MFI) was measured 24 hrs post-infection by flow cytometry.

Next, we tested HAdV17GFP infection efficiencies in receptor-blocking assays in Hela cells. Cells were infected at MOI 100 with representative adenoviruses including HAdV5GFP utilizing CAR as receptor, HAdV3GFP utilizing DSG2 as receptor, and HAdV35GFP entering human cells via CD46 (Fig. 4A). We used recombinant fiber knob proteins for blocking of CAR (HAdV5 knob), CD46 (HAdV35 knob), and DSG2 (HAdV3 fiber-knob). We also produced a recombinant HAdV17 fiber knob protein (HAdV17 knob) to block infection of HAdV17. As shown in Fig. 4A in Hela cells the HAdV3 knob was sufficient in blocking HAdV3, the HAdV35 knob was blocking transduction of a HAdV5/35GFP, and as expected transduction of HAdV5GFP was blocked by the HAdV5 knob but also partially by the HAdV17 knob. Furthermore, we found that the HAdV35 knob could dose-dependently decrease the transduction efficiency of HAdV17GFP from 60% to 10% as measured by determining the percentage of GFP positive cells (Fig. 4B). Interestingly a combination of HAdV5 knob and HAdV35 knob or a combination of 3 blocking reagents (HAdV5 knob, HAdV17 knob and HAdV35 knob) even further reduced transduction efficiencies below 10% (Fig. 4B). Moreover, our experiments revealed that HAdV5- and HAdV17 knobs displayed a moderate and the HAdV35 knob a relatively strong blocking ability, respectively (Fig. 4B), whereas the HAdV3 fiber-knob appeared to have an insignificant effect (not shown). Therefore it can be concluded that in contrast to CAR, CD46 plays a role for HAdV17GFP infection, whereas DSG2 has no role as receptor for HAdV17GFP infection.

Next, we tested HAdV17GFP transduction efficiencies in Hela, EA.hy926- and CHO-C2 cells in the presence of the CD46- and CAR-blocking reagents and the HAdV17 knob generated in this study. In Hela cells the HAdV35 and HAdV17 knobs showed highest blocking efficiencies whereas the HAdV5 knob only showed modest blocking capacities (Fig. 4C). In EA.hy926 cells both HAdV17GFP and HAdV35GFP infections were efficiently inhibited by the HAdV35 knob (from ≥80% to ≤60%) (Fig. 4D). HAdV17GFP infection efficiencies in CHO-C2 cells showed lower transduction efficiencies than HAdV35GFP and HAdV35 knob treatment decreased the percentage of GFP positive cells after HAdV17GFP infection from 52% to 33%, and after HAdV35GFP infection from ~30% to ~3% (Fig. 4E). However, the HAdV17 knob in general was less efficient in inhibiting HAdV17GFP infection of CHO-C2 cells (data not shown) and it displayed a moderate inhibitory effect on HAdV17GFP infection in Hela cells (Fig. 4C). We speculated that the recombinant HAdV17 knob could only moderately block CD46- and the CAR receptors on Hela cells to reduce viral binding. This notion is further supported by the fact that HAdV17GFP was less efficient than HAdV5GFP in transducing CHO-V1 cells (Fig. 3A) and that HAdV5GFP infection in Hela cells was less efficiently blocked by HAdV17 knob as compared to the HAdV5 knob (Fig. 4A). It is of note that all fiber competition experiments described here were performed at 37 °C. However, we observed a similar trend for fiber blocking activities after incubation in cold media (Supplementary Fig. 5) further supporting our findings regarding HAdV17-receptor binding. In summary we concluded that HAdV17GFP infection preferentially depends on CD46 rather than CAR.

### HAdV5 pseudotyped with HAdV17 fiber can increase transducing ability in endothelium cells

**Figure 5:**
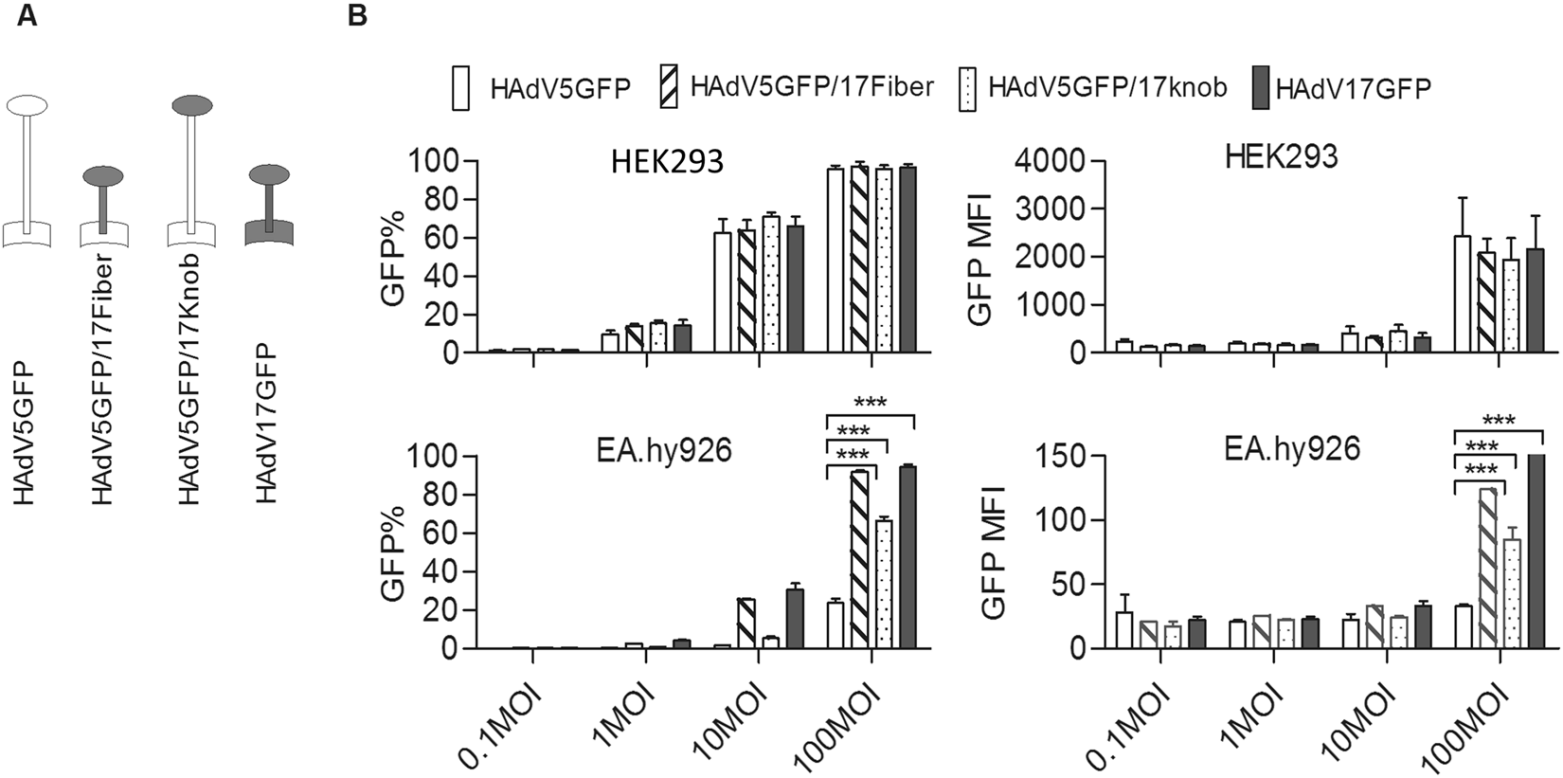
Gain of function study to validate endothelium tropism that is dependent on interaction between CD46 and HAdV17 fiber. (**A**) Schematic representation of chimeric fiber proteins incorporated into HAdV5 capsid and structure of chimeric fiber genes (knob, shaft and tail). The white fiber is derived from HAdV5GFP and the black fiber from HAdV17GFP. HAdV5GFP/17knob contained the shaft and tail from HAdV5 and knob from HAdV17, whereas HAdV5GFP/17fiber contained the tail from HAdV5 and both shaft and knob from HAdV17. **(B)** HEK293- and EA.hy926 cells were transduced with HAdV5GFP, HAdV5GFP/17fiber, HAdV5GFP/17Knob and HAdV17GFP at various multiplicities of infection (0.1, 1, 10 or 100). GFP expression levels were analyzed 24 h post-infection by FACS. Uninfected cells (negative controls) were used to set the background gate at approximately 1%. Percentages provided indicate number of GFP-positive cells (left panel). MFI represented mean fluorescent intensity (right panel). Data points represent mean standard error out of 3 independent experiments (n = 3). ***P < 0.001.

As shown in Fig. 2, EA.hy926-, SKOV3-, and HUVEC cells were refractory to HAdV5GFP infection mainly because of undetectable or low CAR expression. Furthermore the CD46 competitor efficiently blocked HAdV17GFP infection in EA.hy926 cells (Fig. 4D), indicating the CD46 plays a prominent role for HAdV17GFP infection in endothelial cells. To gain further insight into which capsid component of HAdV17 is responsible for interacting with CD46 on EC, we generated chimeric fiber viruses based on HAdV5GFP. Here either the full-length fiber or the knob-coding domain of HAdV5-fiber was replaced by the corresponding sequences of HAdV17 (Fig. 5A). HAdV5GFP/17knob only contained the knob domain from HAdV17 and had the other capsid proteins from HAdV5, whereas HAdV5GFP/17Fiber contained the whole fiber from HAdV17, respectively. To measure transduction efficiencies in EA.hy926 cells we infected these cells using the same virus dose of HAdV5GFP, HAdV17GFP, HAdV5GFP/17Fiber, and HAdV5GFP/17knob and measured the percentage of GFP positive cells. Transduction efficiencies of fiber-modified HAdV5 vectors in HEK293- and EA.hy926 cells were visualized 48 hrs post-infection based on GFP expression (Supplementary Fig. 6). We observed that HAdV5GFP/17Fiber increased the transduction in EC relative to HAd5GFP to levels achieved with HAdV17GFP, while HAdV5GFP/17knob was superior to HAdV5GFP but less infectious than HAdV5/17Fiber and HAdV17GFP (Fig. 5B). Thus, complete replacement of HAdV5 fiber with HAdV17 fiber significantly improved transducing ability of HAdV5GFP in EA.hy926 EC.

### HAdV17 displays broad tropism in CD46 transgenic mice

**Figure 6:**
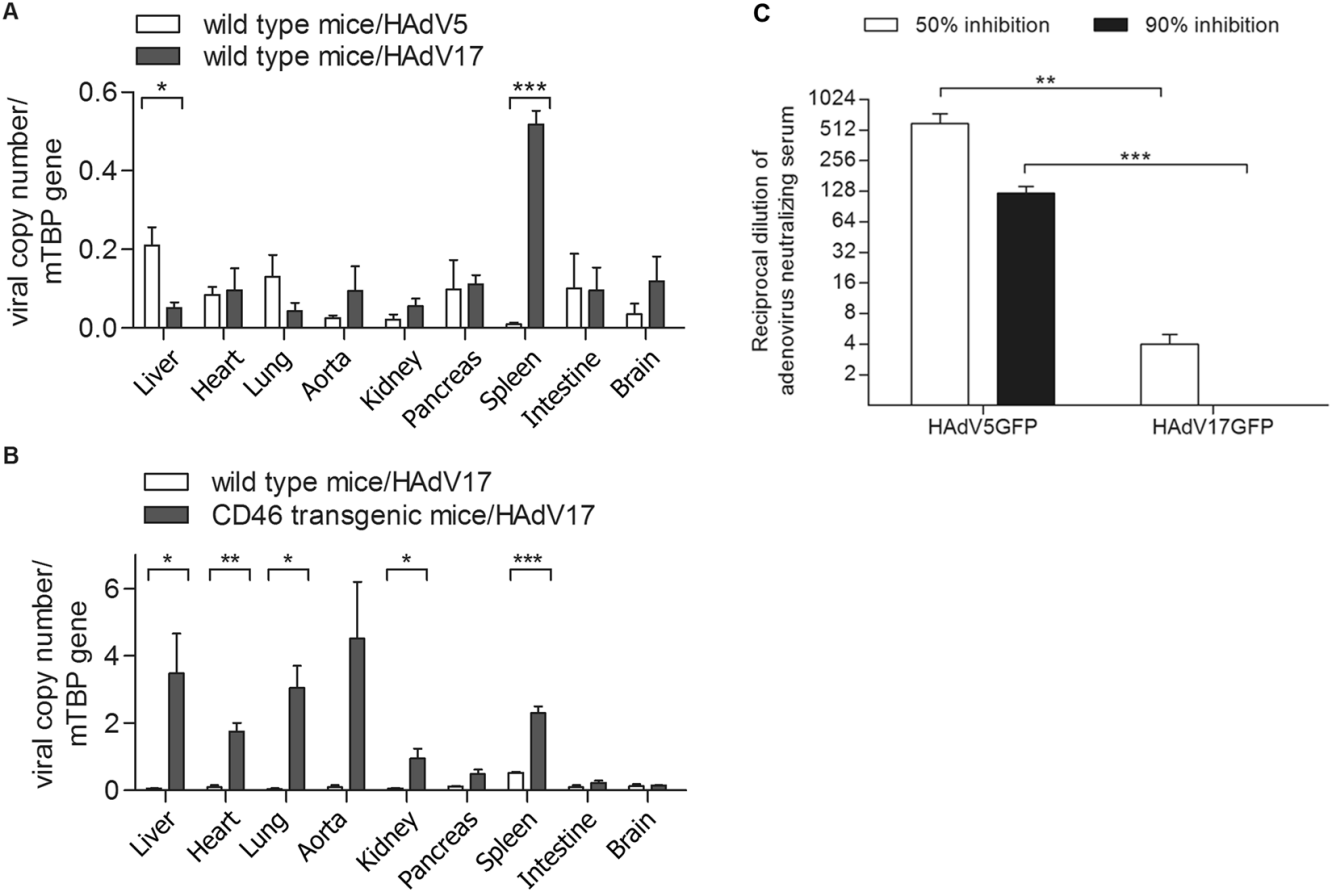
*In vivo* biodistribution study of HAdV17GFP vectors. Viral genomes were measured by qPCR in liver, heart, lung, artery, kidney, pancreas, spleen, intestine and brain 72hrs after systemic administration. 2 × 10e9 transducing units (TU) per mouse of HAdV17GFP were administered intravenously into CD46 transgenic mice and wild type C57BL/6 mice, and the same viral dose of HAdV5GFP was injected into wild type mice serving as control (n = 3 mice per group). **(A)** Direct comparison of HAdV17GFP and HAdV5GFP in C57CBl/6 wild type mice. **(B)** Direct comparison of HAdV17GFP in CD46 transgenic and wild type mice. *P < 0.05, **P < 0.01, ***P < 0.001. **(C**) Neutralizing antibody assay. Reciprocal dilution of dog serum, immunized with HAdV5, was incubated with HAdV17GFP and HAdV5GFP. The serum-virus mixture was used to infect HEK293 cells and 24 hrs post-infection GFP expression levels were determined. **P < 0.01, ***P < 0.001.

To gain insights into the *in vivo* tropism of HAdV17 infection, we compared the viral distribution pattern of HAdV5GFP and HAdV17GFP in C57BL/6 wild type mice by quantifying the viral load in selected organs. Previous reports demonstrated that HAdV5 was sequestered mainly by liver including mostly hepatocytes due to FX-mediated interaction with hexon variable regions^33^. Here we found that fewer HAdV17GFP genome copy numbers were detected in livers of HAdV17GFP injected wild type mice compared to HAdV5GFP transduced mice (Fig. 6A). Furthermore the viral load expressed as viral genome copy numbers of HAdV17GFP C57BL/6 wild type mice was drastically increased in spleen when directly compared to HAdV5GFP injected mice (Fig. 6A). Next we directly compared the biodistribution pattern in C57BL/6 wild type mice and CD46 transgenic mice after systemic administration of HAdV17GFP. Viral genome copy number analyses revealed that significantly increased numbers of genome copy were measured in CD46 transgenic mice compared to wild type mice in the majority of analyzed organs (liver, heart, lung, aorta, kidney, pancreas, and spleen) (Fig. 6B). Highest viral genome copies were observed in aorta, liver, lung, heart, and spleen. This observation further supports our hypothesis that HAdV17 utilizes CD46 as receptor.

An important limitation of HAdV5 in clinical usage is the high seroprevalence (up to 90%) of pre-existing neutralizing antibody eliminating most of the therapeutic vector from circulation. To evaluate the suitability of HAdV17GFP as a novel therapeutic vector, we performed neutralization assays based on one dog serum obtained from a dog immunized with HAdV5. Results revealed that there was low cross-reaction between HAdV5 and HAdV17, as the HAdV5-immunized dog serum poorly neutralized HAdV17GFP (Fig. 6C). We observed 50% inhibition of HAdV5- and HAdV17-derived GFP expression after incubation at a serum dilution of 1:512 and 1:4, respectively (Fig. 6C).

**Figure 7:**
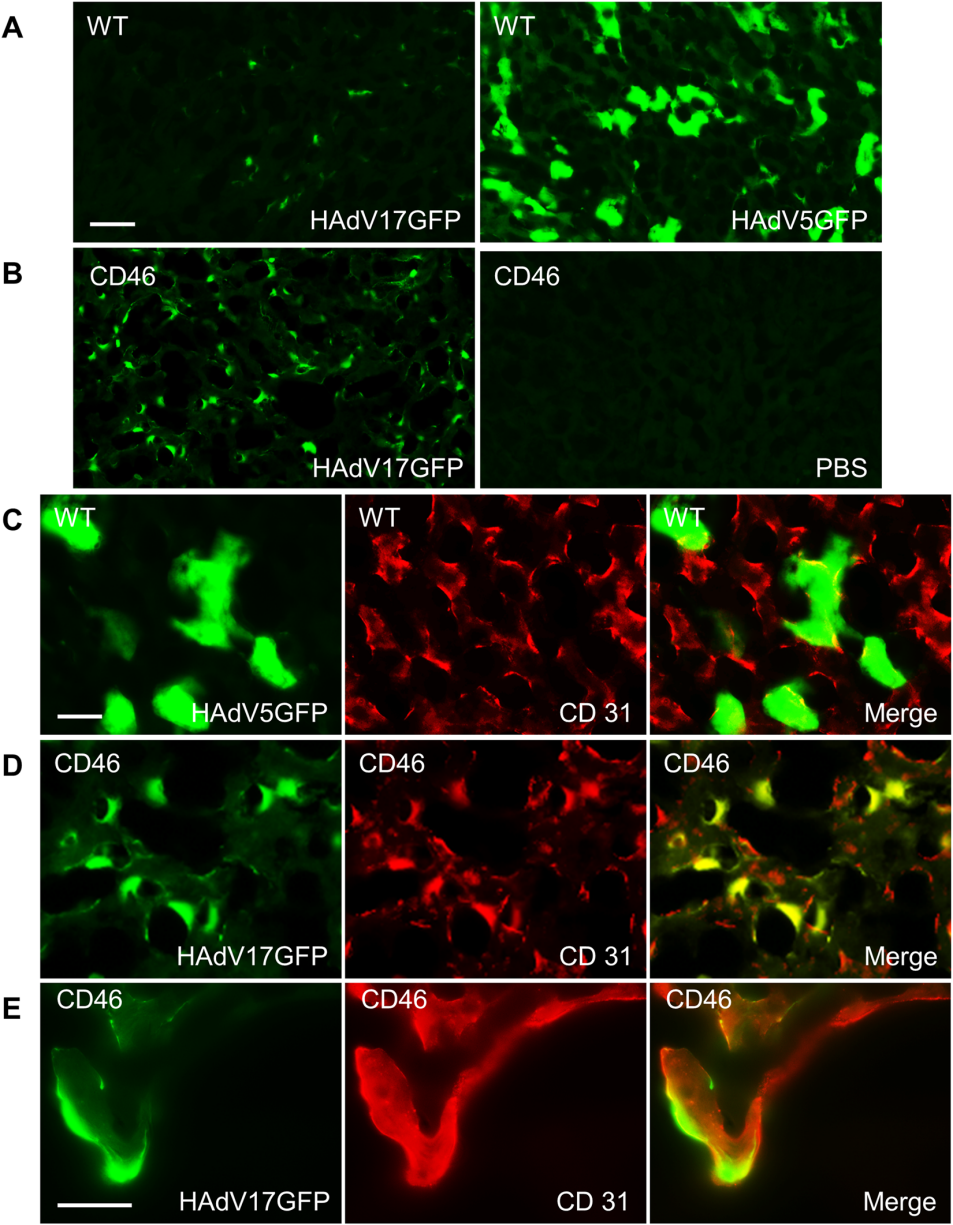
Immunofluorescence analysis of liver sections. Mice were sacrificed 3 days after vector injection. **(A)** HAdV17GFP injection induced weak GFP signals in liver sections of C57BL/6 wild type (WT) mice (left), comparing to HAdV5GFP injection (right). **(B)** HAdV17GFP injection induced high GFP signals in liver sections of CD46 transgenic mice (left), but no detectable signal after PBS injection (right). **(C)** There are no obvious colocalization of GFP and CD31 in liver sections of WT mice after HAdV5GFP injection. **(D**) GFP signals were highly colocalized with CD31 in liver sections of CD46 transgenic mice injected with HAdV17GFP. (**E)** High magnification of GFP signals with CD31 in liver sections of WT mice after HAdV5GFP injection. Scale bars: A-B, 50 µm; C-D, 20 µm; E, 25 µm.

Next we performed histological analyses in liver sections of C57BL/6 wild type (WT) mice transduced with HAdV5GFP or HAdV17GFP. We detected considerably increased GFP fluorescence in sections of HAdV5GFP-transduced WT mice (Fig. 7A right) as compared to sections of HAdV17GFP-transduced WT mice (Fig. 7A left). These data further support our hypothesis that HAdV17 displays a reduced liver tropism compared to HAdV5.

We then analyzed the distribution of HAdV17GFP in liver sections of CD46 transgenic mice. Interestingly, we observed that the GFP fluorescence in HAdV17GFP-transduced CD46 transgenic mice (Fig. 7B left) was more intense than in WT mice (Fig. 7A left). As expected, no GFP fluorescence was detected in CD46 transgenic mice treated with PBS as a control **(**Fig. 7B right).

We then performed immunostainings in order to determine cell types transduced by HAdV5GFP or HAdV17GFP. In liver sections of HAdV5GFP-transduced WT mice we found no obvious co-localization of GFP and CD31, a marker of endothelial cells **(**Fig. 7C**)**. Notably, in sections of HAdV17GFP-transduced CD46 transgenic mice GFP and CD31 were highly co-localized at low **(**Fig. 7D**)** and high magnification (Fig. 7E). In conclusion, these results indicate that HAdV17 unitizes CD46 as receptor and can transduce endothelium cells *in vivo*.

## Discussion

Our recently introduced techniques for cloning and genetic manipulation of large double-stranded DNA virus genomes^19,29^ will expedite the study of the complete human adenoviral spectrum. Here we began with HAdV17 to establish that HAdV17 can transduce EC and primary HUVECs *in vitro*, both of which are refractory to infection with HAdV5. A previous study showed that HAdV49, HAdV35, HAdV5 pseudotyped with fiber from HAdV35 also displayed endothelium tropism *in vitro* and *ex vivo*^10^. Notably, high titres (10,000 viral particles (vps) per cell) of HAdV49 transduced up to 40% primary ECs. In contrast we show for both immortalized ECs and primary HUVECs a large number of cells (80%) transduced by HAdV17 at a significantly lower virus dose (2000 vps/cell), rendering our virus type superior to previously published candidates. However, it remains to be analyzed by a site-by-site comparison which of the adenovirus types is most efficient in transducing endothelia.

We found that HAdV17 can transduce ECs *in vitro* and that this virus efficiently infects CD46 transgenic mice. However, one limitation of our study is the fact that a direct comparison with a predominantly CD46 using adenovirus such as HAdV35 is lacking. However, a previous study demonstrated that a luciferase encoding HAdV35-based vector predominantly transduced lung^34^. We observed robust transduction of liver, lung, and aorta after administration of HAdV17. Note that we quantified vector genome copy numbers per cell for biodistribution studies and Greig and colleagues^34^ measured luciferase expression levels. In the future a direct comparison of HAdV35 and HAdV17 in CD46 transgenic mice should be performed using the identical detection method to analyze the biodistribution pattern.

Fiber protein protruding from the surface of the capsid mediates first attachment to target cells. Here we speculated that fiber from HAdV17 is involved in cell entry, and therefore we constructed chimeric HAdV5GFP/17knob and HAdV5GFP/17fiber viruses. HAdV5GFP/17fiber gained the capability of transducing EC *in vitro* with efficiencies comparable to HAdV17GFP, whereas chimeric virus with the HAdV17 knob resulted in reduced transduction efficiencies of ~50% supporting our hypothesis.

A previous study demonstrated that transduction efficiencies closely correlate with the density of the targeting receptor on the cell surface *in vitro*^35^. Zabner and colleagues showed that HAdV17 displayed robust infection of airway epithelia and neurons but the molecules involved in entry remained unknown^23^. Non-phagocytic liver sinusoidal endothelial cells are thought to capture HAdV5 vectors by pinocytosis in a process that may involve scavenger receptor expressed on endothelial cells (SREC-I)^36^. In contrast, in our study, we have experimental support that a high affinity CD46 inhibitor could block transduction of HAdV17 by 50%, while the HAdV17 knob could block mostly HAdV35 transduction.

To shed further light on receptor usage we also performed sequence alignment and phylogenetic analyses of HAdV11 (uses CD46 and DSG2 as receptor), HAdV35 (uses CD46 as receptor), HAdV37 (uses sialic acid as receptor and binds to CAR and CD46), and HAdV17 (analyzed in this study) (Supplementary Fig. 7). Amino acid alignments indicated that HAdV17 may also use CD46 and CAR as attachment receptors. However, here we could confirm that CD46 plays a role in cell entry of HAdV17 and that CAR most likely plays a negligible role, and therefore in silico data (Fig. 1) were not confirmed. Note that the theoretical isoelectric point (pI) of HAdV17 calculated using the pI/Mw tool within the ExPASy Proteomics server was relatively high which was also shown for AdV37 (Supplementary Fig. 7). This may indicate that charge may play a role in virus-cell surface interaction, although here we found that there was no interaction of HAdV17 with sialic acid. Therefore, in contrast to HAdV37 which uses the sialic acid-containing glycan GD1a as receptor, this could not be confirmed for HAdV17.

CD46 is a cofactor member expressed on all nucleated cells which may enable HAdV17 and the chimeric vector HAdV5/17fiber to infect more diverse cell types and tissues such as stem cells, epithelial cells, and neurons. However, our data implicate that HAdV17 seems not to be a strong binder of CD46 which may also explain why the analyses of amino acid sequences revealed no conserved amino acids based on X-ray diffraction experiments compared to HAdV35 and HAdV11 which are strong CD46 binders. Although we provide evidence that CD46 plays a role in cell entry, it remains to be elucidated whether another cell surface protein which may contribute as a synergistic effect to HAdV17 cell entry. For instance some other molecules such as integrins or coagulation factor X could represent potential candidate receptors. A previous study demonstrated that infectivity of fiber-pseudotyped Ad5 viruses in which the fiber was derived from several subgroup D viruses (Ad47, Ad33, Ad24, Ad45, Ad17, Ad30) strongly depends on binding to the coagulation factor X^37^.

In conclusion we introduce and characterize a novel adenovirus species D derived vector which can transduce endothelial cells and that CD46 and not CAR or sialic acids play a role for cell entry. However, further studies are required to further analyze the mode of action when entering the cell and whether an unknown receptor exits.

## Material and Methods

### Cell lines

Human epithelial kidney cell line HEK293 (ATCC CRL-1573), human epithelial lung carcinoma cell line A549, human liver cancer cell line Huh7, SKHep-1, and the immobilized human umbilical vein cell line EA.hy926 (ATCC CRL-2922) were cultured in Dulbecco’s minimal essential medium (PAN-Biotech, Germany) supplemented with 10% fetal bovine serum (PAN-Biotech, Germany), penicillin (100 U/ml), streptomycin (100 g/ml), and 2 mM glutamine. The culture medium for Huh7 cells was supplemented with non-essential amino acids (PAN-Biotech, Germany). CHO-C2, CHO-K1, CHO-V1, CHO-E606, and Jurkat cells (ATCC TIB-152) were cultured in RPMI 1640 medium supplemented with 10% FBS and antibiotics (PAN-Biotech, Germany). The primary human umbilical vein cell line (HUVEC, Promo Cell C-12203) was cultured in serum-free Promo Cell Growth Media (Germany). MMDH3 cells were cultured in RPMI 1640 with 10% FBS, EGF (55 ng/ml), IGF II (16 ng/ml), Insulin 10 µg/ml and antibiotics.

### Vector construction

As described previously the whole adenovirus genomic DNA of HAdV17 was directly cloned into the plasmid p15A by linear-linear homologous recombineering (LLHR) to generate p15A-AdV17^19^. The first generation vector p15A-HAdV17GFP with E1 gene deletion replaced by eGFP was generated by modifying p15A-HAdV17 through linear-circular homologous recombineering (LCHR) based on ccdB selection^29^. Briefly, the ccdB-Amp cassette encoding ccdB and ampicillin was flanked by 50 bp homology arms flanking the E1 gene by PCR using the primers 17HR1for and 17HR1rev. Then 500 ng purified PCR product and 500 ng p15A-HAdV17 were co-transformed into GBred-gyrA462 electro-competent bacteria after competent cells were induced by 0.2% arabinose for 40 minutes at 37 °C. Restriction digestion analysis was performed to select ampicillin-resistant clones containing the ccdB-Amp cassette (p15A-HAdV17-ccdB). Next, the CAG-eGFP cassette with homology arms flanking the E1 gene was amplified using primers 17HR2for and 17HR2rev and introduced into GB05-red competent cells together with p15A-HAdV17-ccdB to generate p15A-HAdV17GFP. The same strategy was used to construct plasmid p15A-FGAdV5GFP which functioned as a control. To generate chimeric vectors, we used similar procedures as described above. Briefly, the ccdB-Amp cassette including 50 bp flanking homology sequence derived from knob- or fiber- coding sequence of HAdV5 was inserted into p15A-HAdV17, which was subsequently replaced by knob- or fiber- coding sequence of HAdV17 to generate p15A-HAd5VGFP/17knob and p15A-HAdV5GFP/17fiber.

For extraction of complete viral genomes from HAdV17 and HAdV5 purified virions, viral particles were lysed by adding lysis buffer (proteinase K, 0.5% SDS, 10 mM EDTA, 10 mM Tris-HCl, pH 8.0), incubated at 56 °C for 4 hours and then treated with RNaseA (Sigma Aldrich) for 30 min. Protein debris were removed by phenol-chloroform extraction. The extracted RNA-free viral DNA was precipitated with ethanol and eluted in dH_2_O. Detailed information on the cloning procedure and primers used can be obtained upon request. For primer sequences please refer to Supplementary Table 1.

### Generation of stable E1 expressing cell lines for HAdV17 vector production

We found that HAdV5 E1-expressing HEK293 cell lines failed to rescue HAdV17GFP virus (data not shown), thus we established stable HAdV17 E1-encoding cell lines based on HEK293 and A549 cells. Briefly, the E1-coding sequence from HAdV17 was cloned into pIRESneo3 vector (Clontech) to generate the plasmid pCMV-HAdV17E1-IRES-neo. Linearized pCMV-HAdV17E1-IRES-neo was transfected into HEK293 and A549 cells by FuGENE (Promega). Single cell clones were selected using G418 as selection marker. PCR and RT-PCR were performed to check the genome integration of the plasmid and expression of HAdV17-derived E1. To verify somatic integration of the E1 transgene, we used primers 17E1for and 17E1rev resulting in a PCR product of 2808 bp in length. RT-PCR was performed using primers RTfor and RTrev. We included a negative control without reverse transcriptase in the experimental setup. HAdV17GFP was successfully reconstituted in the HEK293 based cell line stably expressing E1 from HAdV17. Further information on the cloning procedure and primer sequences used for these cloning steps can be obtained upon request. For primer sequences please refer to Supplementary Table 1.

### Virus amplification and purification

Wild type HAdV5 strain was obtained from the American Type Culture Collection (ATCC). HAdV17 was a clinical isolate obtained from the Department of Virology at the Max von Pettenkofer Institute at the Ludwig-Maximilians-University Munich. To amplify wild type viruses, 70–80% confluent A549 cells (for amplification of HAdV17) and HEK293 cells (for amplification of HAdV5) maintained in twenty 15-cm tissue culture dishes were infected by virus at a multiplicity of infection (MOI) of 1 to 3. After complete cytopathic effect (CPE) had occurred, infected cells were harvested following by four freeze-thaw cycles. Virus purification and storage were prepared as described previously^38^. Briefly, viruses were purified by CsCl gradients to remove cell debris and empty viral particles. After PD-10 column desalting dialysis, viruses were stored at −80 °C. Chimeric first generation viruses HAdV5GFP/17knob and HAdV5GFP/17fiber were amplified in HEK293 cells using the same protocol. The E1-deleted adenoviral vector HAdV17GFP was amplified in a stably HAdV17-E1 expressing cell line which was generated in this study.

### Titration experiments

Transducing units (TUs) of virus preparations were determined by quantitative real-time PCR (qPCR) detecting GFP and the housekeeping gene hB2M using primers GFPfor, GFPrev, hB2Mfor and hB2Mrev. For primer sequences please refer to Supplementary Table 1. Briefly, HEK293 cells at 90% confluency grown in 6-well plates were infected with varying volumes (0.1 µl, 0.5 µl, 2.5 µl) of purified virus using serum-free medium. At 2 hours post-infection, cells were harvested with trypsin (0.05%; PAN-Biotech) and washed with DPBS to remove extracellular virus particles. Total DNA was purified by phenol/chloroform extraction and subsequent ethanol precipitation. Quantitative real-time PCR was performed and analyzed to determine relative copy number of virus genomes in cells. We calculated the TUs according to the copy number of virus vector genomes and the number of cells that were infected initially. The calculation of each of the multiplicities of infection (MOI) used in this study was based on the titers of virus stocks expressed in TUs. Furthermore the physical titer of virus particles was determined as described earlier^38^.

### Quantification of viral genomes by quantitative real-time PCR (qPCR)

HEK293-, A549-, Hela- and EA.hy926 cells were infected at MOIs of 0.1, 1, 10, and 100 with HAdV5GFP and HAdV17GFP in 24-well plates for 2 h. Cell collection and total cellular DNA extraction were performed as described above. Quantitative real-time PCR was performed using the C1000 Touch™ Real-Time PCR Detection System (Bio-Rad). The PCR was based on the following program: pre-incubation/activation at 95 °C for 5 min, amplification and data collection during 40 cycles (95 °C for 15 s and 60 °C for 30 s). The SYBERGreen master mix (ThermoScientific) was used for these PCR reactions.

In order to detect genomic copy number of virus replication at different time points, real time PCR was performed 2, 10, 24, 48, 72 hours post-infection (if not otherwise stated). Cellular DNA was isolated from 3 independent samples at each time point and virus replication and particle production were monitored.

### Analyzing transduction efficiencies *in vitro*

To detect transduction efficiencies in different cell lines, fluorescence-activated cell sorting (FACS) was carried out. 1.0 × 10^5^ cells were seeded in 24-well plates. After full confluency was reached, cells were infected with varying MOIs (0.1, 1, 10, 100) and incubated overnight. 24 hrs later cells were washed once with PBS and detached with trypsin-EDTA. Cells were resuspended in Dulbecco’s minimal essential medium containing 10% FBS, centrifuged (1500 g, 3 min), and washed in DPBS before they were fixed with 4% formaldehyde. Fluorescence profiles were obtained by analyzing 10,000 viable cells by use of a flow cytometer and CellQuest software (BD Biosciences). Background signal were obtained by analyzing the negative control which were uninfected cells. The percentage of GFP-expressing cells was determined by selecting a region of fluorescence above the background of auto-fluorescence from uninfected cells.

### Immunostaining

To detect CAR (coxsackie and adenovirus receptor) expression on the cell surface of different cell lines using flow cytometry, HEK293, A549 Hela EA.hy926 were counted and 0.5 × 10^6^ cells were washed with PBS supplemented with 1% BSA, centrifuged (1500 g, 3 min), and resuspended in 100 μl PBS/BSA and 2.5 μl anti-CAR antibody (Santa Cruz, sc-56892). Following an incubation step at 4 °C for 1 hour cells were washed again with PBS/BSA, to remove unbound antibodies and resuspended in 100 μl PBS/BSA containing 0.5 μl of an APC labeled goat anti-mouse secondary antibody (Santa Cruz, sc-3818). After incubation for 1.5 hours at 4 °C with continuous shaking, cells were again washed with PBS/BSA and finally resuspended in 400 μl PBS for flow cytometry using FACS (BD). As controls each cell line was also incubated without primary antibody. In all experiments 10,000 viable cells were counted. The CD46 antibody (Mouse anti-human CD46 monoclonal antibodyIgG2B, FAB2006B, R&D systems) was used to detect surface expression of CD46 on different cell lines, and secondary donkey anti-mouse antibody is conjugated with Alexa Fluor® 568 (ThermoFisher, A10037).

### Production and elution of recombinant knob proteins

Recombinant knob proteins derived from adenovirus types 5, 35, and 3 (HAdV5-, HAdV35-, HAdV3 knobs, respectively) were previously described^6,39,40^. Recombinant HAdV17 knob was produced as followed. The HAdV17 knob domain (HQ910407, NCBI) was synthesized from Genescript and ligated into the plasmid pQE30 (Qiagen). Then the HAdV17 knob protein encoding plasmid was transformed into *E*. *coli* bacteria and grown overnight in 800 ml of LB medium. Recombinant 17 knob were expressed in *E*. *coli* cells which were subsequently lysed, purified on NI-NTA columns (Qiagen). In the next step the solution was loaded onto a polypropylene column (Qiagen). After the solution passed the column by gravity and washed with 50 ml of wash buffer, 10 ml of elution buffer were added and 1 ml fractions of purified protein were collected. To control the fractions and the size of the protein, recombinant proteins were verified by a SDS-PAGE gel (Bio Rad gel, at 140Vfor 45 min).

To determine the final protein concentration the Bradford protein assay was performed. A BSA standard was pipetted with concentrations from 1 mg/ml–0.0625 mg/ml. The protein sample was used in three ratios (sample: water) undiluted, 1:2, and 1:5. Protein assay dye was used at a ratio of 1:5 (dye/water). Then 20 μl of the different protein solutions were added to a micro cuvette. After the dye was added and a homogenous solution was formed, samples were read at 596 nm using UV Vis. Protein concentrations were determined based on a respective standard curve.

### Competition assays

Cells were seeded in 48-well plates until 90% confluency was reached. Then cells were pre-incubated with recombinant fiber knobs at room temperature for 1 hr applying different concentrations (1.25, 2.5, 5, 10, 20 µg/ml). Then GFP labeled adenovirus was added at MOI 100 per cell in 200 µl growth media for 2 hrs. After that cells were washed and GFP expression was monitored by FACS (BD) after 24 hrs.

#### Depletion of sialic acids on the cell surface

The experiment was performed in concordance to the publication by Lenman *et al*.^41^ using neuraminidase (sialidase) from Vibrio cholera. A549 cells were seeded on 24-well plates, washed three times with serum-free medium, and 200 µl/well of sialidase solution (10mU/200 µl) was added before incubation for 1 h at 37 °C. After washing three times with serum-free medium and refilling with 200 µl serum free medium cells were incubated at 4 °C for 10 min, virus added at desired concentrations and incubate for 1 hour on ice. After three washing steps with serum-free medium in order to remove unbound virions, 500 µl growth medium was added followed by an incubation step at 37 °C and 5% CO_2_ for 24 hours. After detaching cells using trypsin and one washing step with PBS, 500 µl formalin (2%) were added and incubated for 15 min at room temperature. After washing twice with PBS, FACS analyses to measure eGFP expression were performed.

### *In vivo* biodistribution studies

All *in vivo* experiments were approved by the local Ethics Committee for Animal Research at the University of Washington and were performed in accordance with relevant guidelines and regulations. 2 × 10^9^ viral transducing units of HAdV17GFP dissolved in DPBS were administered by tail-vein injection into three C57BL/6 wildtype mice and three CD46 transgenic mice. For PCR analyses, mice were euthanized 72 hrs after virus administration, blood was flushed from the circulation with 5 ml of PBS, and tissues were collected and rapidly frozen in liquid nitrogene. Genomic DNA was isolated from approximately 0.1 g tissue and analysed by qPCR. For direct comparison 2 × 10^9^ transducing units of HAdV5GFP were tail-vein injected into three wildtype mice and as controls PBS-treated CD46 transgenic mice and wild type mice were used.

For immunostaining, mice were euthanized 72 hrs after virus administration; tissues were rapidly collected and fixed in 4% paraformaldehyde for 24 hrs. Tissues were then incubated in 30% sucrose overnight, embedded in optimal cutting temperature medium (OCT)-containing cryomolds (Tissue-Tek), and frozen at −80 °C. 16 µm sections were cut from embedded tissues, rinsed in phosphate-buffered saline (PBS) for 5 minutes three times and permeabilized in PBST (PBS + 0,1% Triton X-100) for 5 minutes. For immunostaining sections were blocked in PBS containing 3% bovine serum albumin (BSA) and 10% normal goat serum (NGS) for 1 hr. The primary antibody (rat anti-CD31 antibody, BD bioscience 557355) was diluted 1:1000 in PBS with 3% BSA, and incubated for 1 hr at room temperature. The secondary antibody (goat anti-rat AF555, Invitrogen A21434) was diluted 1:800 in PBS + 3% BSA, and incubated for 1 hr at room temperature. Then slides were mounted with Fluoromount-G (Biozol), covered with coverslips, and stored at 4 °C. Immunofluorescence was captured using a fluorescence microscope Nikon ECLIPSE Ni-U (20X objective) and monochrome images were acquired through fluorescence filters for FITC and TRITC. Images were acquired and merged using the software Nikon NIS.

### Neutralizing antibody assay

For cross reaction studies we used serum from a pre-immunized dog injected with a high-capacity adenoviral vector based on HAdV5 as described previously^42,43^. Briefly, serial dilution of heat-inactivate serum were produced and mixed with an MOI of 10 for HAdV5GFP and HAdV17GFP. Infection was performed in a 96 well plate (Luminometer compatible plate). This mixture was incubated at 37 °C for 15 min. HEK293 cells (1 × 10^4^) diluted in growth medium were then added to the serum-virus mixture. 24 hours later, medium was removed and lysis buffer from the GFP Quantitation Kit (Cell Biolabs, AKR-120) was added. After 15 min the incubation plate was directly transferred into the fluorescence plate reader at Ex/Em = 488 nm/507 nm. Each serum-virus well was analyzed in triplicate. Titration curves were plotted with the mean values and standard deviations from triplicate samples.

### Sequence alignments

Multiple sequence alignments were performed by Clustal Omega^44^ using the following sequences derived from the protein database at NCBI^45^: HAd37 (GI:315583499), HAdV12 (GI:49258423), HAdV1 (GI:202957874), HAdV8 (GI:190356629), HAdV4 (GI:202957893), AdV40 (GI:202958295), HAdV21 (GI:294662464), HAdV11 (GI:126031349), HAdV3 (GI:542373661), HAdV14 (GI:215261396), HAdV35 (GI:189339610), HAdV26 (GI:383291041), HAdV48 (GI:383291055), HAdV17 (GI:202957963).

### Statistical analyses

All experiments were performed using at least triplicates. Statistical significance was determined by unpaired Student’s *t*-test using GraphPad Prism 5. All data are reported as mean +/− SEM (standard error of the mean). Statistical comparison was performed using the two-tailed student’s test and a value of *p* < 0.05 was considered to be relevant compared to the respective control group.

## Electronic supplementary material

Supplementary Information
