# Supplementary material for: Human adenovirus type 17 from species D transduces endothelial cells and human CD46 is involved in cell entry
Source: Sci Rep. 2018 Sep 7;8:13442. doi: 10.1038/s41598-018-31713-x (PMC6128842; doi:10.1038/s41598-018-31713-x)
Supplement: Supplementary file 1 — Supplementary Information [file 41598_2018_31713_MOESM1_ESM.pdf]

Supplementary Information

Human adenovirus type 17 from species D transduces endothelial cells and human CD46 is involved in cell entry

Jing Liu,[a*] Philip Boehme,[a†§] Wenli Zhang,[a§] Jun Fu,[b,c] Roma Yumul,[d] Kemal Mese,[a] Raphael Tsoukas,[a] Manish Solanki,[a‡] Michael Kaufmann,[e] Ruirui Lu,[f±] Achim Schmidtko,[f±] A. Francis Stewart,[c] André Lieber[d], Anja Ehrhardt[a#]

Institute for Virology and Microbiology, Center for Biomedical Education and Research (ZBAF), Witten/Herdecke University, Witten, Germany[a]; Shandong University-Helmholtz Institute of Biotechnoloy, State Key Laboratory of Microbial Technology, School of Life Science, Shandong University, Jinan, 250100, People's Republic of China[b]; Genomics, Biotechnology Center, Technische Universität Dresden, BioInnovations Zentrum, Dresden, Germany[c]; University of Washington, Department of Medicine, Division of Medical Genetics, Seattle, USA[e]; Division for Medical Biochemistry, Center for Biomedical Education and Research (ZBAF), Witten/Herdecke University, Witten, Germany[d]; Institute for Pharmakology and Toxicology, Center for Biomedical Education and Research (ZBAF), Witten/Herdecke University, Witten, Germany[f]

Running Title: Human CD46 leads to endothelial tropism of adenovirus

[#]Address correspondence to Anja Ehrhardt, anja.ehrhardt@uni-wh.de

[§] Philip Böhme and Wenli Zhang contributed equally to this work and were listed in alphabetical order.

[*]Present address: Department of Oncology and cancer immunotherapy, University of Maryland School of Medicine, Baltimore, MD, USA

†Present address: Medical Student, Department of Human Medicine, Faculty of Health, Witten/Herdecke University, Witten, Germany

[±]Present address: Institute of Pharmacology, College of Pharmacy, Goethe University, Frankfurt am Main, Germany

[‡]Present address: Institute for Experimental Gene Therapy and Cancer Research (IEGT), Medical University Rostock, Rostock, Germany

Supplementary Figure legends

Supplementary Fig. 1. Schematic outline virus vectors generation. (A) Flowchart of p15A- and ccdB- based homologous recombineering to construct plasmid p15A-HAdV17GFP. The whole adenovirus genomic DNA of HAdV17 was directly cloned into plasmid p15A-HAd17ITR to generate p15A-HAdV17 by linear-linear homologous recombineering (LLHR). Subsequently, linear-circular homologous recombineering (LCHR) was performed to construct the first generation E1-deleted adenoviral HAdV17 vector. In brief, PCR was first performed to amplify HR1-ccdB-Amp, for which p15A-ccdB-Amp served as PCR template. For homologous recombination 50bp of homologous regions (17HR1) were introduced by using primers containing these homology arms. After diagnostic restriction analysis to verify integrity of the intermediate clone p15A-AdV17-ccdB, the PCR product of HR2-CAG-eGFP amplified from pCAGeGFP was transformed into GB05-red arabinose-induced electrocompetent cells together with p15A-HAdV17-ccdB. Restriction analysis and sequencing were performed to verify cloning of the final vector p15A-AdV17GFP. (B) Diagram of p15A- and ccdB- based homologous recombineering to construct the plasmids p15A-HAdV5GFP, p15A-HAd5GFP/17knob and p15A-HAd5GFP/17fiber. p15A-HAdV5GFP was constructed using the same method as described in subfigure (A). Subsequently the knob or fiber region of p15A-HAdV5GFP was initially replaced with the PCR product containing the selection marker ccdB and the ampicillin resistance gene. Next the ccdB cassette was replaced by the PCR product amplified from HAdV17 knob or fiber region resulting into the final constructs p15A-HAd5GFP/17knob and p15A-HAd5GFP/17fiber. Engineered adenoviral plasmids were amplified and purified by ultracentrifugation.

Supplementary Fig. 2. Generating stable HAdV17-E1 expressing complementing cell lines based on HEK293- and A549 cells. (A) The E1 cassette from HAdV17 (2808bp) was amplified by PCR and ligated into the multiple cloning site of pIRESneo3 resulting into the plasmid pCMV-HAdV17E1-IRES-neo. **(B)** Methylene blue staining was performed to mark the positive cell clones after completing the selection procedure. **(C)** PCR and RT-PCR were performed to analyze presence of the stable transduced plasmid pCMV-HAdV17E1-IRES-neo and E1 expression in single cell clones. For the PCR a product of 850 bp was expected. For the RT-PCR a PCR product of 756 bp was expected and the negative control was performed without reverse

transcriptase. N: negative control **(D)** Amplification of HAdV17GFP was monitored at each passage (P0 to P3) by expression of the reporter gene GFP.

Supplementary Fig. 3. HAdV17 has tropism for endothelium cells *in vitro*. (A) Cell line screening *in vitro*. HEK293-, A549-, Hela-, Huh7-, MMDH3-, and EA.hy926 cells were transduced with HAdV5GFP and HAdV17GFP at various MOIs (0.1, 1, 10 or 100). GFP expression levels were analyzed 24 hrs post-infection by FACS analyses and the mean fluorescence intensity (MFI) measured. Uninfected cells (negative controls) were used to set the background gate at approximately 1%. A total of 10,000 viable cells were counted.

Supplementary Fig. 4. Flow cytometry analyses to detect cell surface expression levels of CAR and CD46 on various cell lines. (A) 0.5×10^6 HEK293-, A549-, Hela-, EA.hy926-, MMDH3-, and HCT116 cells were counted and incubated with the anti-CAR antibody followed by an incubation step with an APC labeled goat anti-mouse secondary antibody. As negative controls each cell line was also incubated without supplementation of the primary antibody. **(B)** 1×10^6 EA.hy926-, Hela-, SKOV3-, CHO E606 and CHO-C2 cells were counted and incubated anti-CD46 antibody followed by the Alexa Fluor® 568 conjugated donkey anti-mouse secondary antibody. As negative controls each cell line was also incubated without supplementation of the antibody. On the *y* axis the percent max (the cell count in each bin divided by the cell count in the bin that contained the largest number of cells) is shown. The *x* axis is the fluorescence intensity in log scale.

Supplementary Fig. 5. Fiber blocking assays in various cell lines performed in cold media. Cells were pre-incubated with increasing concentrations of respective recombinant fiber knob proteins in cold media and then exposed to infection with HAdV5GFP and/or HAdV17GFP. Uninfected cells (negative controls) were used to set the background gate and the percentage of GFP positive cells was determined. **(A)** CHO-C2 were pretreated with HAdV35 knob at increasing concentrations at 4°C for 1 hour, and then exposed to HAdV17GFP with 20,000 viral particles (vp) per cell. GFP expression was measured 48 hrs post-infection (p.i.) by flow cytometry. **(B)** CHO-CAR cells were pretreated with HAdV5 knob at increasing concentrations at 4°C for 1 hour, and then exposed to HAdV17GFP with 10,000 using vp per cell. GFP expression was measured 48 hrs post-infection by flow cytometry. **(C)** HeLa cells were

pretreated with HAdV5 fiber knob, HAdV17 knob, or a combination of HAdV5 and HAdV35 knobs at increasing concentrations at 4°C for 1 hour. Subsequently cells were exposed to HAd17GFP using 1000 vp/cell and GFP expression levels were measured 24 hrs post-infection by flow cytometry.

Supplementary Fig. 6. Transduction efficiencies of fiber-modified HAdV5 vectors visualized in HEK293- and EA.hy926 cells. HEK293- and EA.hy926 cells were transduced with HAdV5GFP, HAdV5GFP/17fiber, HAdV5GFP/17Knob and HAdV17GFP at MOI of 100. GFP expression levels and cytopathic effects (CPE) were captured 48 hrs post-infection.

Supplementary Fig. 7. Analyses of the adenovirus fiber knob. (A) Phylogenetic analysis of adenovirus fiber knob. The phylogenetic tree was constructed using Clustal W tool based on the neighbour joining method. **(B)** The theoretical isoelectric point (pI) was calculated using the pI/Mw tool within the ExPASy Proteomics Server.

Supplementary table 1. Primers used in this study.

17HR1 for	5'–TGCCAGCGAGTAGAGATTTCTCTGAGCTCCGCTCCCAGAGTGTGAGAAAATTTGTTTATTTTTCTAAATAC–3'
17HR1rev	5'–GTGCCCCTTCTGGCGCCCAGGTGCATGTGGCACTTGATCAGCATGTTATTTTATATTCCCCAGAACATCAGG–3'
17HR2 for	5'–TGCCAGCGAGTAGAGATTTCTCTGAGCTCCGCTCCCAGAGTGTGAGAAAAGCTCTAGCCCCTAGTTATTAATAG–3'
17HR2 rev	5'–GTGCCCCTTCTGGCGCCCAGGTGCATGTGGCACTTGATCAGCATGTTATTCCTATACAGTTGAAGTCGGAAG–3'
5HR1 for	5'–CCAGCGAGTAGAGTTTTCTCCTCCGAGCCGCTCCGACACCGGGACTGAAATTTGTTTATTTTTCTAAATAC–3'
5HR1 rev	5'–CACCCCCCTCCTGTTACCCAAATGCAAGGAACAGCGGGTCAGTATGTTATGTTATATTCCCCAGAACATCAGG–3'
5HR2 for	5'–CCAGCGAGTAGAGTTTTCTCCTCCGAGCCGCTCCGACACCGGGACTGAAAGCTCTAGCCCCTAGTTATTAATAG–3'
5HR2 rev	5'–CACCCCCCTCCTGTTACCCAAATGCAAGGAACAGCGGGTCAGTATGTTATGCCTATACAGTTGAAGTCGGAAG–3'
knob HR1 for	5'–GCACAGGTGCCATTACAGTAGGAAACAAAAATAATGATAAGCTAACTTTGTTTGTTTATTTTTCTAAATAC–3'
knob HR1 rev	5'–GAAAAATAAACACGTTGAAACATAACACAAACGATTCTTTATTCTTGGGCTTATATTCCCCAGAACATCAGG–3'
knob HR2 for	5'–GCACAGGTGCCATTACAGTAGGAAACAAAAATAATGATAAGCTAACTTTGTGGACAACACCAGACACATCTC–3'
knob HR2 rev	5'–GAAAAATAAACACGTTGAAACATAACACAAACGATTCTTTATTCTTGGGCTTATTGTTGGGCAATATAGGAG–3'
fiber HR1 for	5'–TTTCCTCCTGTTCCTGTCCATCCGCACCCACTATCTTCATGTTGTTGCAGTTTGTTTATTTTTCTAAATAC–3'
fiber HR1 rev	5'–GAAAAATAAACACGTTGAAACATAACACAAACGATTCTTTATTCTTGGGCTTATATTCCCCAGAACATCAGG–3'
fiber HR2 for	5'–TTTCCTCCTGTTCCTGTCCATCCGCACCCACTATCTTCATGTTGTTGCAGGTCCTGTCACTCAAACTGGC–3'
fiber HR2 rev	5'–GAAAAATAAACACGTTGAAACATAACACAAACGATTCTTTATTCTTGGGCTTATTGTTGGGCAATATAGGAG–3'
17E1for	5'–CGGGATCCCGATGAGACACCTGCGCCTCCTG–3'
17E1rev	5'–CGGGATCCCGCTAATCTGTGTCCTCCCCACTG–3'
RT for	5'–GATACGATGAGACCAAGTCCA–3'
RT rev	5'–TCTTATACACGTGGCTTTTGG–3'
GFP for	5'–CAAGATGAAGAGCACCAAAGG–3'
GFP rev	5'–TAGGTGCCGAAGTGGTAGAAG–3'
hB2M for	5'–TGCTGTCTCCATGTTTGATGTATCT–3'
hB2M rev	5'–TCTCTGCTCCCCACCTCTAAGT–3'
Universeprobe-HADV	5'-[FAM] CCGGGTCTGGTGCAGTTTGCCCGC [BHQ1]–3'
HAdV-C qPCR-fwd	5'–CAGGACGCCTCGGAGTACCTGA–3'
HAdV-C qPCR-rev	5'–GGCGCCACCGTGGGGTT–3'
HAdV-D qPCR-fwd	5'–CAGGACGCCTCGGAGTACCTGA–3'
HAdV-D qPCR-rev	5'–GGGCCACCGTGGGGTTC–3'

Supplementary Fig. 1, Liu et al.

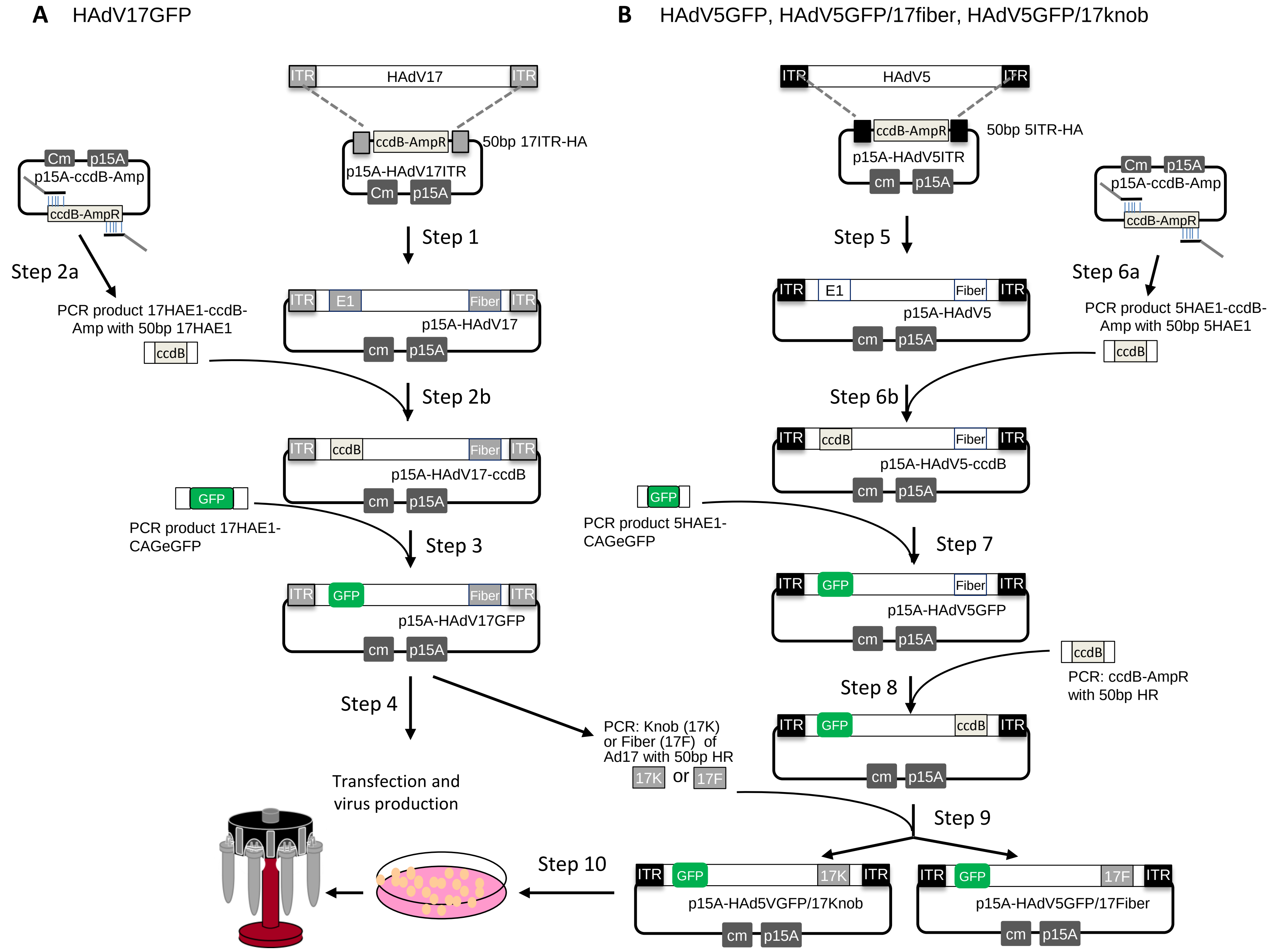

Supplementary Fig. 2, Liu et al.

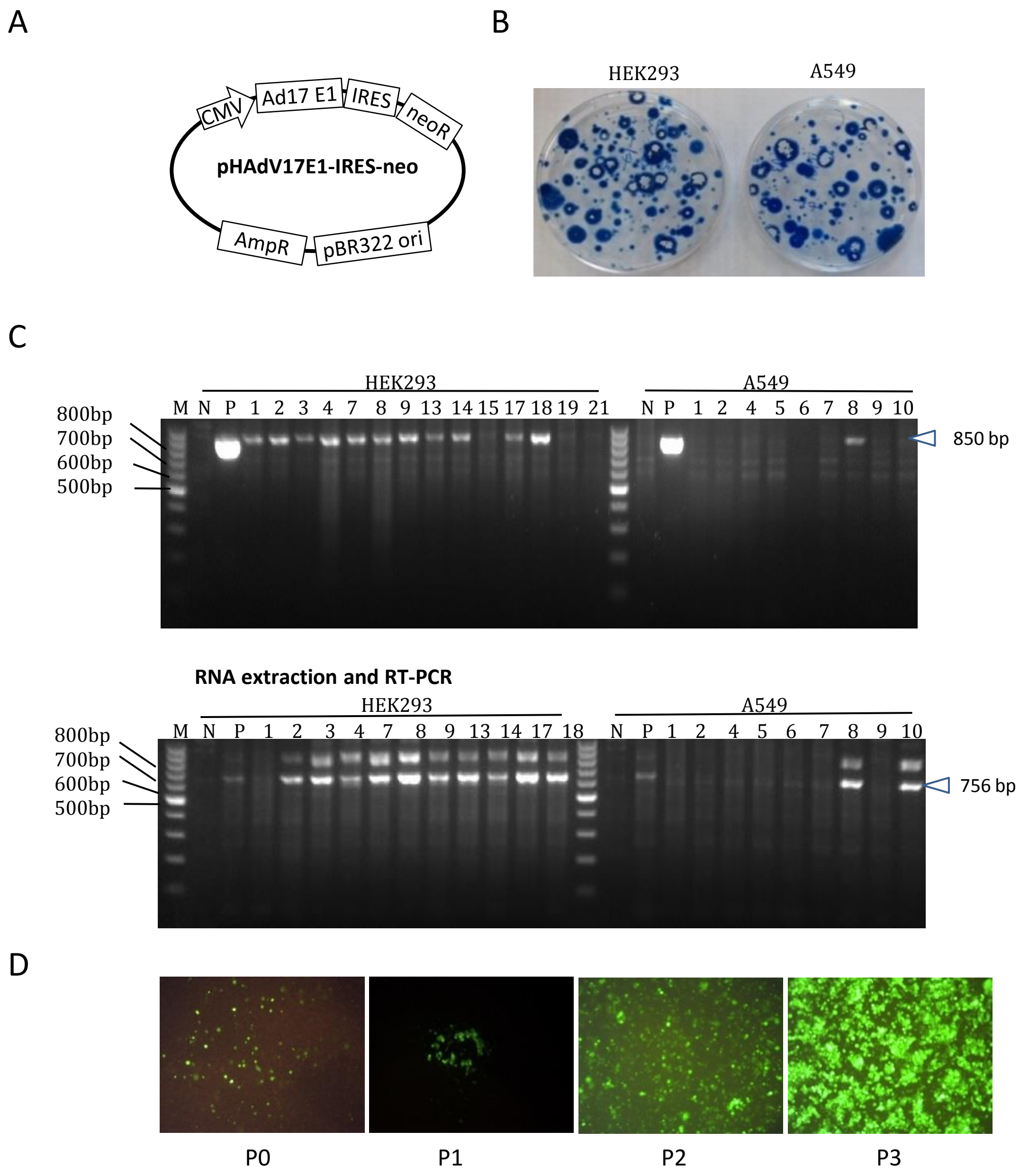

Supplementary Fig. 3, Liu et al.

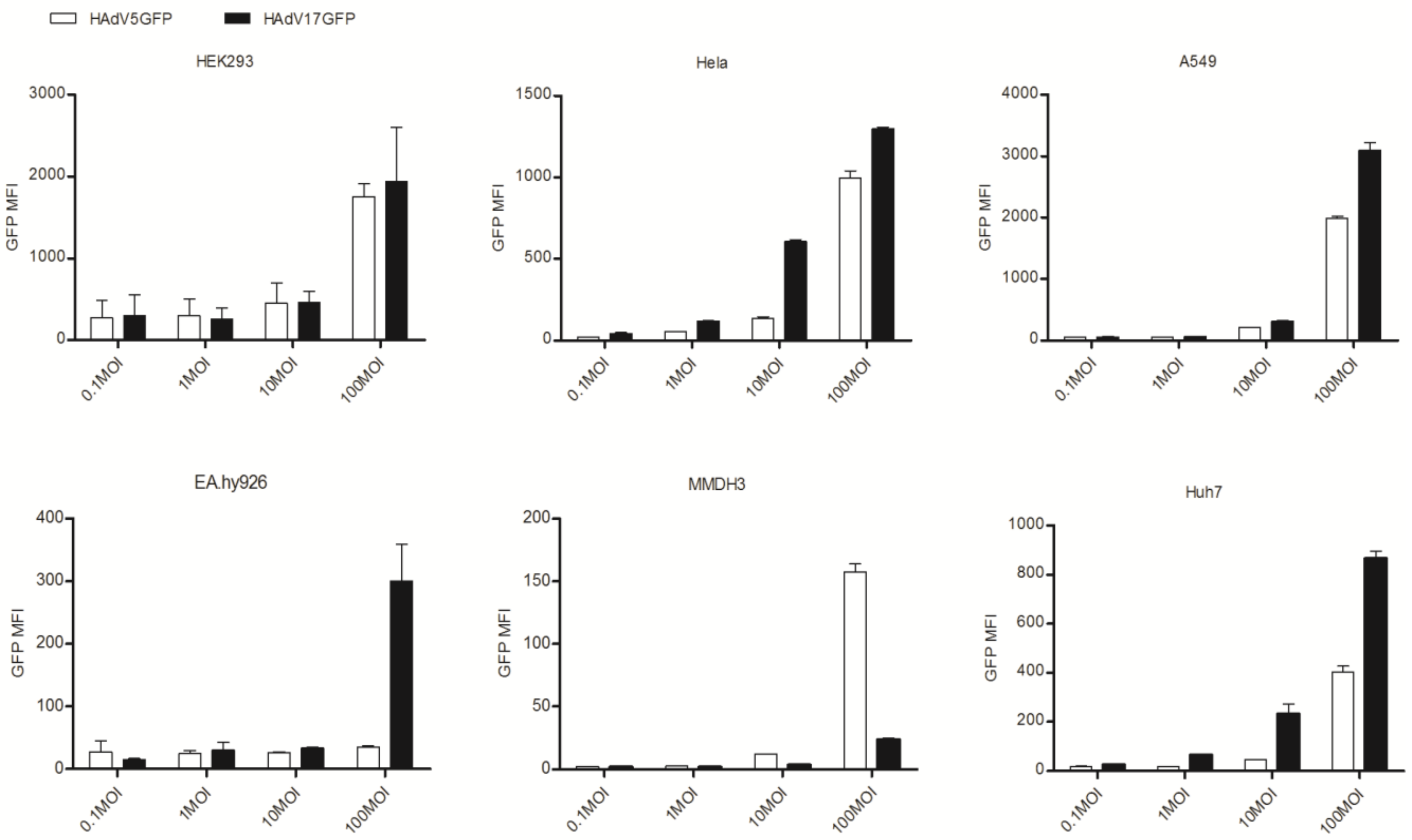

Supplementary Fig. 4, Liu et al.

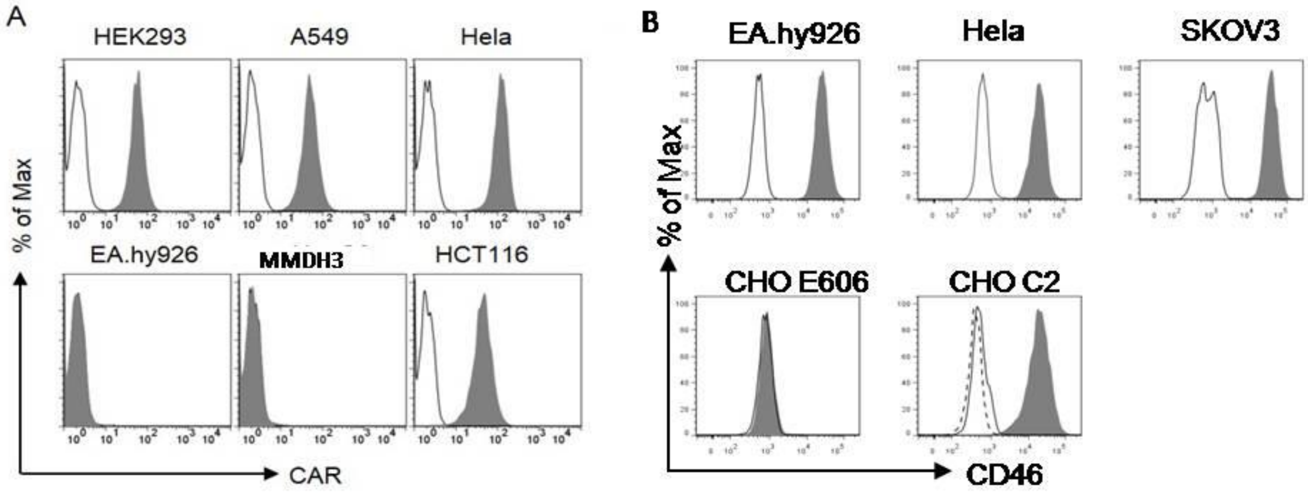

Supplementary Fig. 5, Liu et al.

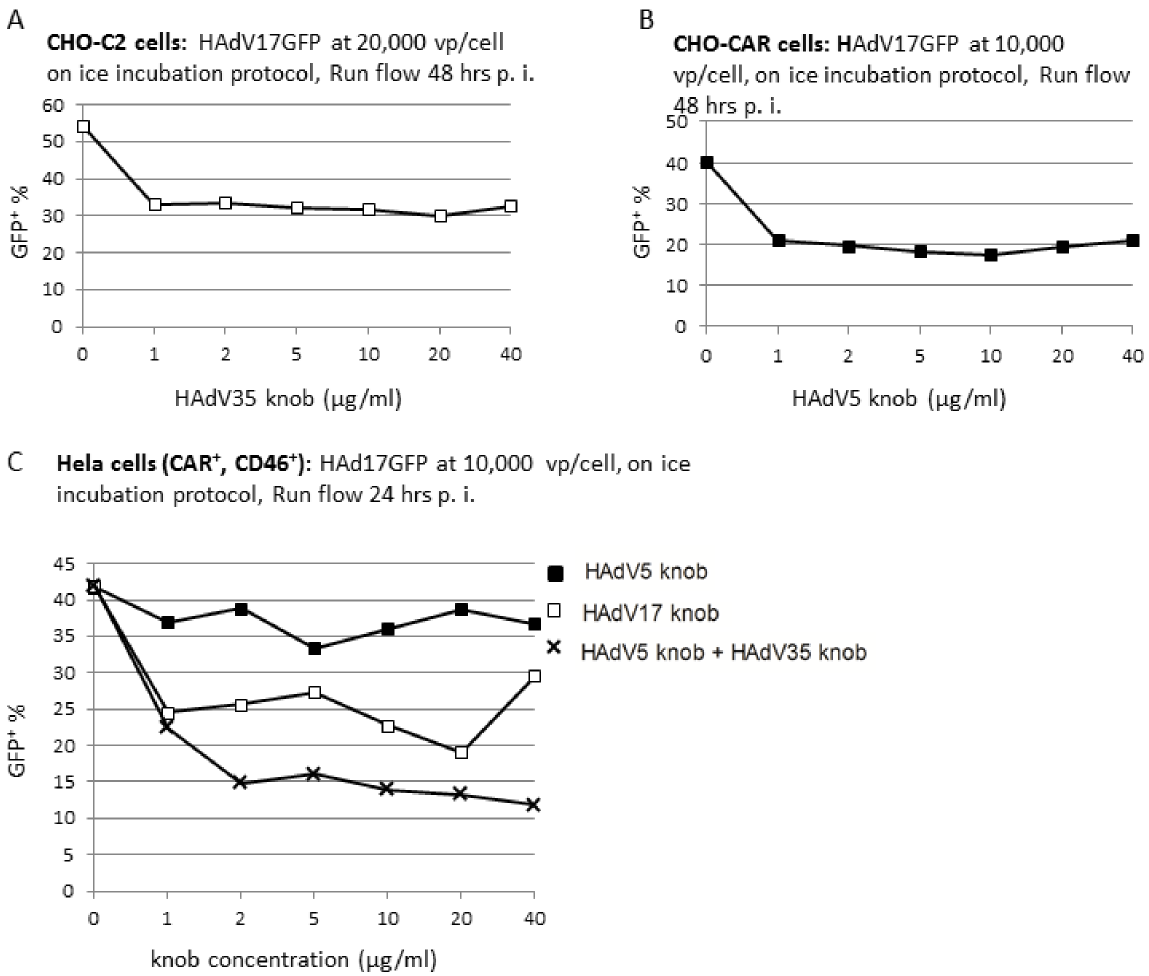

Supplementary Fig. 6, Liu et al.

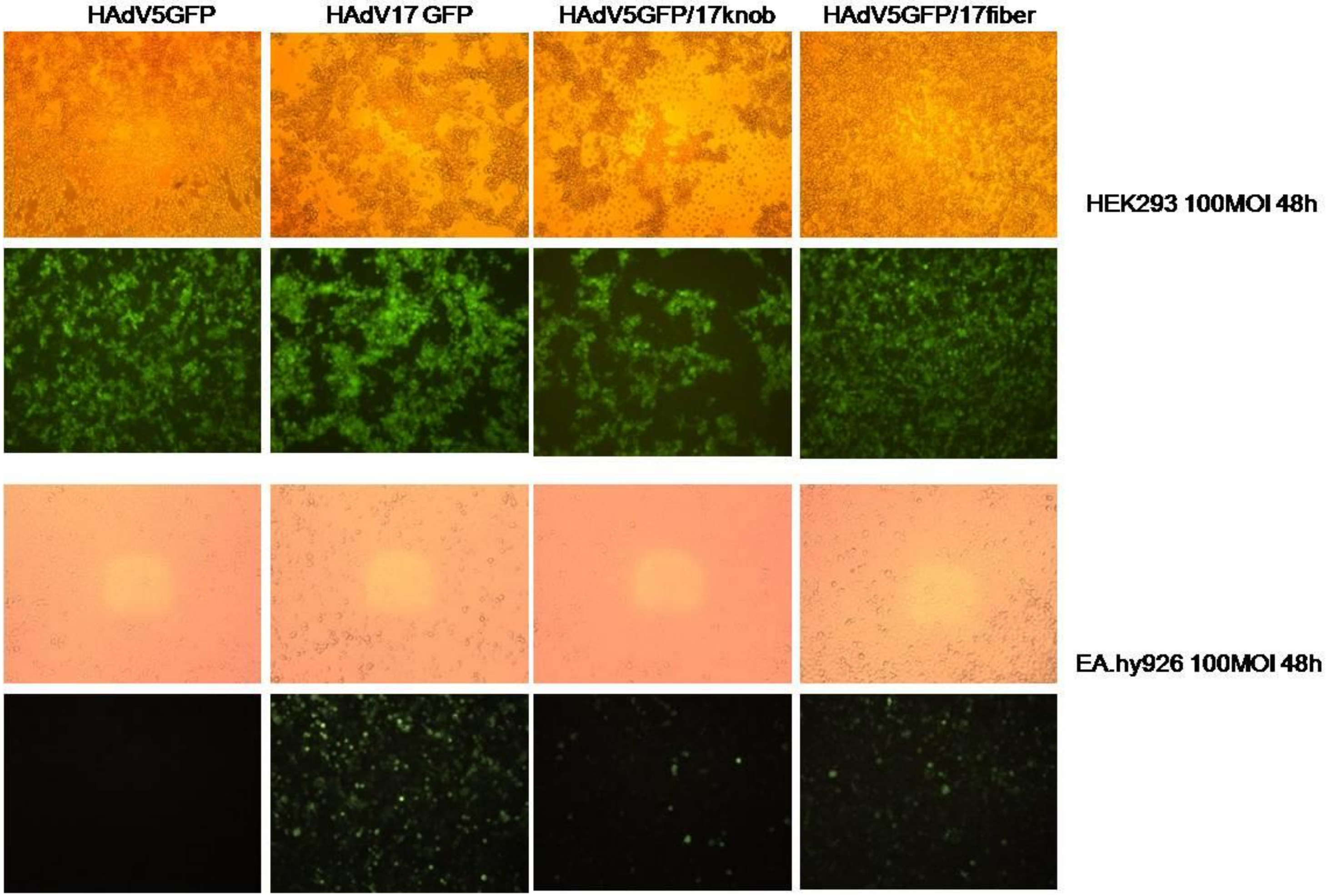

Supplementary Fig. 7, Liu et al.

A

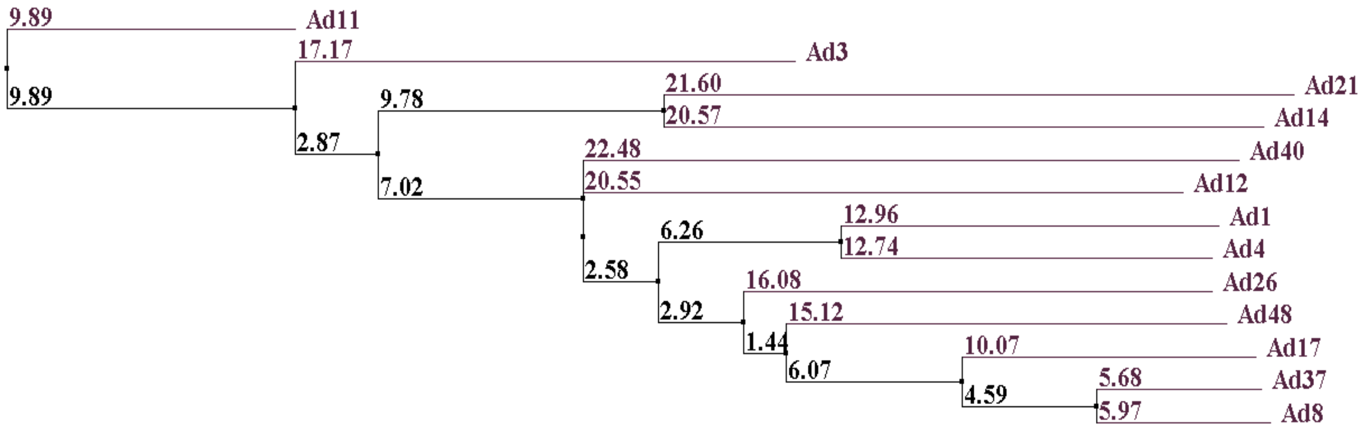

B

Theoretical pI	AdV5	AdV35	AdV37	AdV17
Fiber knob	5.88	4.66	9.14	7.85
Hexon	4.39	4.42	4.54	4.46
Penton	4.58	4.78	4.71	5.15
